# Supplementary material for: Engineering broad-spectrum inhibitors of inflammatory chemokines from subclass A3 tick evasins
Source: Nat Commun. 2023 Jul 14;14:4204. doi: 10.1038/s41467-023-39879-3 (PMC10349104; doi:10.1038/s41467-023-39879-3)
Supplement: Supplementary file 1 — Supplementary Information [file 41467_2023_39879_MOESM1_ESM.pdf]

SUPPLEMENTARY INFORMATION

Engineering Broad-spectrum Inhibitors of Inflammatory Chemokines from Subclass A3 Tick Evasins

Shankar Raj Devkota[1], Pramod Aryal[1], Rina Pokhrel[1], Wanting Jiao[2], Andrew Perry[3], Santosh Panjikar[1,4], Richard J. Payne[5,6], Matthew C.J. Wilce[1], Ram Prasad Bhusal[1,*] and Martin J. Stone[1,*]

[1]Monash Biomedicine Discovery Institute, and Department of Biochemistry and Molecular Biology, Monash University, Clayton, VIC 3800, Australia

[2] Ferrier Research Institute, Victoria University of Wellington, Wellington 6140, New Zealand; Maurice Wilkins Centre for Molecular Biodiscovery, Auckland 1142, New Zealand

[3]Monash Bioinformatics Platform, Monash Biomedicine Discovery Institute, Monash University, Clayton, VIC 3800, Australia

[4]Australian Synchrotron, ANSTO, Clayton, VIC 3168, Australia

[5] School of Chemistry, The University of Sydney, Sydney, NSW 2006, Australia

[6]Australian Research Council Centre of Excellence for Innovations in Peptide and Protein Science, The University of Sydney, NSW 2006, Australia

*Correspondence: ram.bhusal@monash.edu; Tel.: +61-3-9905-5937;

martin.stone@monash.edu; Tel.: +61-3-9902-9246

(a)

```
IRI601               (IRI)------------------GPAPSAKENEKAPLCL-------------------------PQ   35
EVA-4                (RSA)------------------------------------------EVPQMTSSSAPDLEE   15
EV974                (ACA)-----------------------------------------------------ENTQQ    5
EVA-1                (RSA)----------------------------------------------------------   0
A0A6M2E2L8           (ATL)--------------------------------------------------------EE    2
A0A023FT45/EV985     (APA)----------------------------------------------DEESE---ELGAS   10
A0A6M2E4M9           (ATL)-------------------EPAATAT---PSCLEDPCNSTSSSGEESEEDY---------   29
A0A6M2E316           (ATL)------------AHEEDSHEPAVTGS---ASSLDDTSNRTSASGEEIEEDYDD-------   38
A0A6M2E374           (ATL)----------------DSHEPAVNES---ATSL---NNRTSASGEEIEEDYDD-------   31
A0A6M2E5Z3           (ATL)----------------DSHEPAVNES---ATSL---NNRTSASGEEIEEDYDD-------   31
A0A6M2E879           (ATL)-------------------EPAATAT---PSCLEDPSNSTSSSDASEESESDGTGTSQES   38
A0A6M2E5F6           (ATL)------------------------------------------LQDASRESDDTGTSHES   17
A0A6M2E2U0           (ATL)-------------------------------------------HDASLQTDVTDASLQS   16
A0A023G2M2           (ATT)-------------------------KDL---SSTTEETAVGYSSTTDNINASLGTETTTDQA   34
A0A023G2G7           (ATT)-------------------------KDL---SSTTEETTVGYSSTTDNINASLGTETTTDGT   34
A0A1E1X165           (AAL)----------------------------------------------TDPGNSTECTNTT   13
A0A0C9S461/E1243     (AAM)-------------------------------------------------GSARNHTEDN   10
A0A023FFD0/EV991     (ACA)--------------------------------------------  ENGEGTTQPDYDN   13

IRI601                    ESLINNRDPNGCNYQLLPYFTEDGMGGGFLAIDCSKSCPEGT---HETVVDGNSCVAKVD   92
EVA-4                     --EDDYTAYAPLTCYF-----TNSTLGLLAPPNCSVLCNSTTTWFNETSPNNASCLLTVD   68
EV974                     EEQDYDYG--TDTCPFPV--LANKTN-KAKFVGCHQKCNGG----DQKLTDGTACYVVER   56
EVA-1                     -EDDEDYG-DLGGCPFLV--AENKTG-YPTIVACKQDCNGT----TETAPNGTRCFSIGD   51
A0A6M2E2L8                ALTDDFIENTECACPVQQ--LENANGTVLRAPGCTYFCGTV----SCPIPDDYPCYAVTL   56
A0A023FT45/EV985          TDVDYEELDANCTCPAPA--LTSTRNNKHYPLGCIYNCSSY----NCTIPDGTPCYVLTL   64
A0A6M2E4M9                ----DSIVLGECYCLAPH--LHTNQGNVSRVVGCIYTCEGR----NCTAEEDYPCYDTTL   79
A0A6M2E316                --NYNSGALGGCYCPAAH--LRTRQGNFSRVAGCIYICEER----NCTVEHDYPCYDITL   90
A0A6M2E374                --NYDSAVLGGCYCPAPN--LLNLQGNFSRLAGCVYICGER----NCTVKHDYPCYDITL   83
A0A6M2E5Z3                --NYDSTVLGGCYCPAPN--LLNLQGNFSRLAGCIYICEER----NCTVKHAYPCYDITL   83
A0A6M2E879                NQTDDYYPQAECLCPVFV--LRNTMNSTSKPPGCIYSCESK----NCTIPDGEACYNISF   92
A0A6M2E5F6                NETDDYYPTGGCTCPVIV--LNNPNNSTVKPPGCIYPCGAV----NCTLTDEEPCYNISL   71
A0A6M2E2U0                NETDDYYPTDGCTCPITN--LNNHNSSATKPPGCVYSCGTV----NCTLTDEEPCYNISL   70
A0A023G2M2                TTEYYDYGLENCTCSYPT--LRNSMN-LTATVGCHQICDGI----NCTLPEGTRCYTLQS   87
A0A023G2G7                TTEYYDYELDNCTCSHPT--LRNSMN-VTGAVGCSLPCNGV----NCTLPDGTNCYTLGS   87
A0A1E1X165                EDYDYNYGTLNCSCPVGV--LYNTNGTMPKQVGCEYHCGTE----NCKVPVGTACYDLNI   67
A0A0C9S461/E1243          STEYYDYEEARCACPARH--LNNTNGTVLKLLGCHYFCNGT----LCTAPDGYPCYNLTA   64
A0A023FFD0/EV991          STDYYNYEDFKCTCPAPH--LNNTNGTVMKPIGCYYTCNVT----RCTAPDTYPCYNLTE   67

IRI601                    SLSKEE-------ATVMLGACDKGSCKPENPPQHLTVTLMVEGEEKEE---   133
EVA-4                     FLTQDAILQENQPYNCSVGHCDNGTCAGPPRHAQC-----W----------   104
EV974                     KVWDRMT--PMLWYECPLGECKNGVCEDLRKKEDC-----RKGNGEEK---   97
EVA-1                     EGLRRMT--ANLPYDCPLGQCSNGDCIPKETYEVC-----YRRNWRDKKN-   94
A0A6M2E2L8                ETFAQMA--PRVEHTCLPGICQNGTCVPSANEEAC-----MKGVYIE----   96
A0A023FT45/EV985          GEVKEHLQIGSTVPNCTCGLCRNGTCVSNGTVEEC-----FAVEEIEET--   108
A0A6M2E4M9                EVFYTME--VNLTQECVPGMCRNGTCVTTGEKEEC-----HK---------   114
A0A6M2E316                ELFSAME--VNATQNCFPGMCRNGTCVTTGEKEEC-----FK---------   125
A0A6M2E374                EEFFAME--VNATQDCFPGICRNGTCVTTGEQEEC-----FK---------   118
A0A6M2E5Z3                EEFSAME--VNATQDCFPGICRNGTCVTTGEQEEC-----FK---------   118
A0A6M2E879                VEYKNMK--VNQTQNCLLGSCQNGTCVPDGQEEQC-----YQIEA------   130
A0A6M2E5F6                QAYKLME--INMTQSCPLGTCQNGTCVPNGQEEQC-----YSS--------   107
A0A6M2E2U0                LEFGSME--VNKTQSCLLGTCNNGTCVPDGGKELC-----YLGED------   108
A0A023G2M2                QLASVQLQARSFEHTCQVGVCHNGTCILNGTVEGC-----YPLVPK-----   128
A0A023G2G7                KLTSVQLQARTIEQSCQAGVCHNGTCILNGTVELC-----FPLVPKTKTLS   133
A0A1E1X165                TVVNTW--KTNTTHPCPVGICNGDMCRKNGTTEEC-----FKATF------   105
A0A0C9S461/E1243          QQVRTL--TTYPNTSCAVGVCMKGTCVKNGTMEQC-----FKTP-------   101
A0A023FFD0/EV991          HQAKNL--TTSPTTLCAVGNCDHGICVPNGTKELC-----FKAPNLEE---   108
```

(b)

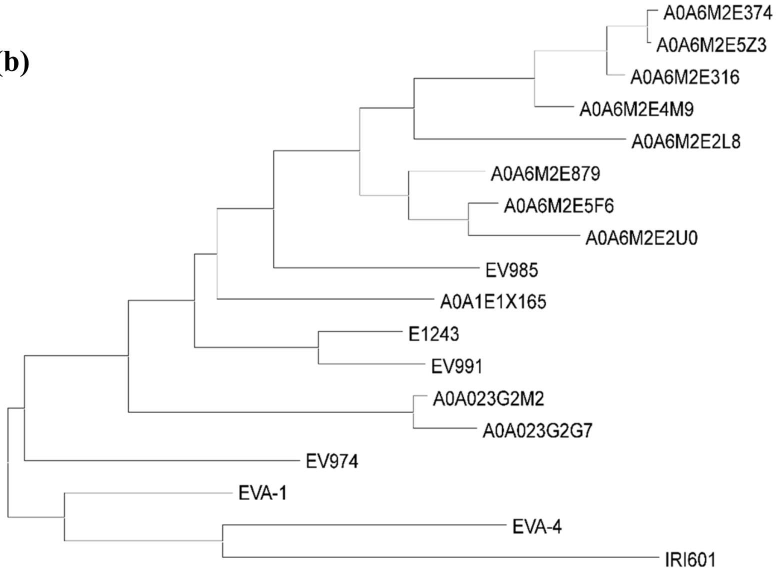

Supplementary Figure 1. Sequences and features of class A3 evasins. (a) Multiple sequence alignment (obtained using Clustal Omega) of the sequences of class A3 evasins,along with the sequences of EVA-P974 (EVP974, class A1), EVA-1 (Evasin-1, class A1), EVA-4 (Evasin-4, class A1) and EVA-IRI601 (IRI601, class A2). Sequence names are the primary accession code found in UniProt. Sequence A0A0C9S461 corresponds to EVA-AAM1001. Conserved cysteines (yellow), additional cysteines (red), conserved glycine (green), putative tyrosine sulfation sites (pink) and putative glycosylation sites (sky blue) are highlighted. Abbreviations used for tick species are *ATL, A. tuberculatum; AAM, A. americanum; ACA, A. cajennense; APA, A. parvum; ATR, A. triste; IHO, Ixodes ricinus and RSA, R. sanguineus.* (b) The unrooted tree (ClustalO, IQ-TREE) showing the relationships between the sequences.

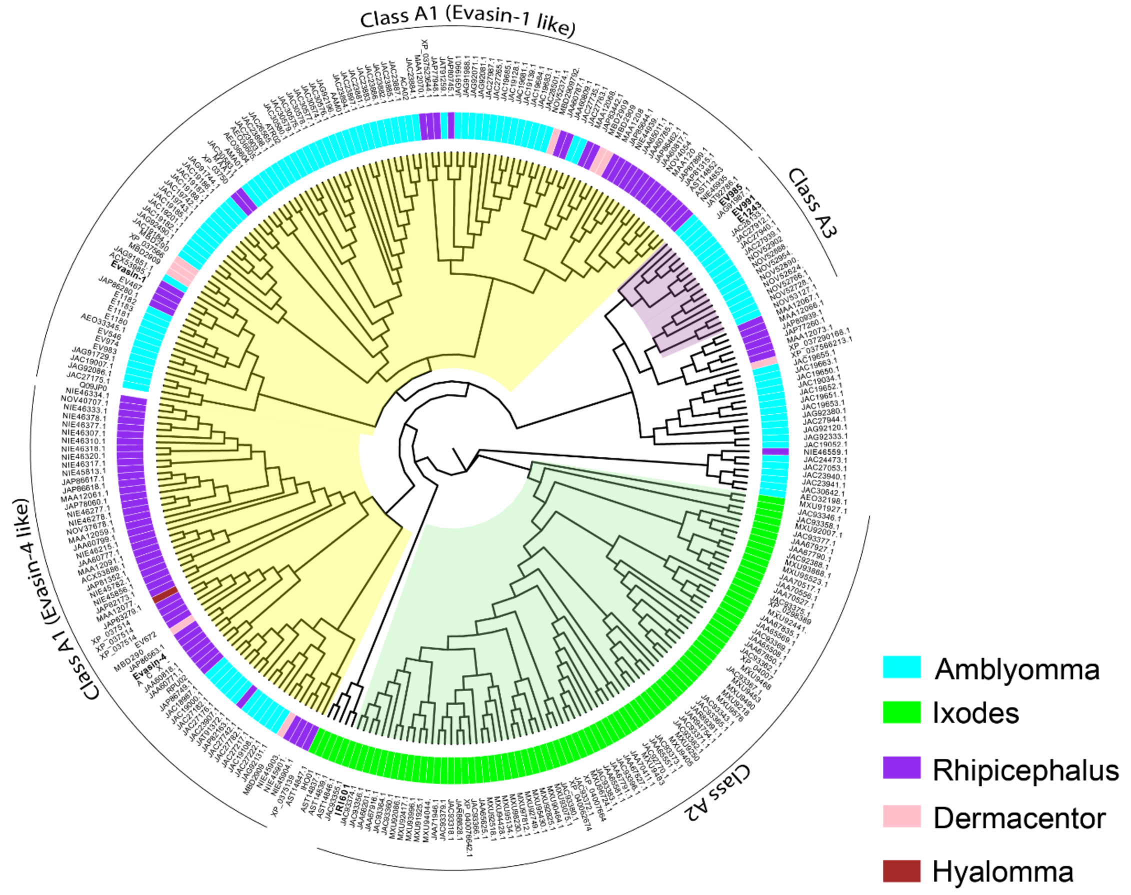

Supplementary Figure 2. Neighbour-joining tree of class A evasin proteins. A midpoint-rooted, neighbour-joining tree represented as a cladogram, based on MUSCLE alignments of the class A evasins. Background colours show subclasses: A1 (light yellow), A2 (green) and A3 (purple). The genus of each node is indicated by coloured segments in the outer ring and shown in the legend. The figure is generated based on the sequences available in *Front. Cell. Infect. Microbiol., 22 October 2021 Sec. Parasite and Host Volume 11 – 2021*.

(a)

```
EV1243/AAM1001 (AAM) --------------RNHTEDNSTEYY--DYEEARCACPARHLNNTNGTVLKLLGCHYFCN     44
JAP80939.1     (RAP) -LSMCNNQAPAESGKNINVKVSISRPDSPVSGTVCTIPVLR----GNSLRRPVGCCHFCR     55
MAA12067.1     (RZA) -LSMCNNQAPAE---------SVSRPDSPVSGTVCSIPVLR----GNSLRRPVGCCHFCR     46
MAA12066.1     (RZA) -LSMCNNQAPAE---------SVSRPDSPVSGTVCSIPVLR----GNSLRRPVGCCHFCR     46
MAA12073.1     (RZA) AVSMCNSPAPAGSGKKISVQVSISRPESRSASTICRIPFLH----GKLTMKPVGCCLFCR     56
JAP77260.1     (RAP) -LSMCNNPAPAESGKKISVQVSISRPESRSASTICRIPFLH----GKFTMKPVGCCLFCR     55
XP_037290168.1 (RMI) -VSMCNNPELAASGKNISVKVSISRPESRSRETVCTIPFLH----GQHTMKPVGCCLLCA     55

EV1243/AAM1001      GTLCTAPDGYPCYNLTAQQVRTLTTYPNTSCAVGVCMKGTCVKNGTMEQCFKTP-----       98
JAP80939.1          DMNETLPDKTPCYAIPPLEALRMKPHARRSCPLGLCENGVCKPTGKYEICESIATIKKM       114
MAA12067.1          DMNETLPDKTPCYAIPPLEALRMKPHARRTCPLGLCENGVCKPTGKYEICETIATIKKM       105
MAA12066.1          DMNETLPDKTPCYAIPPLEALRMKPHARRTCPLGLCENGVCKPTGKYEICETIATIKKM       105
MAA12073.1          EMNRTLQDKTPCYAIPPHEALQMKLRAPRSCPLGLCENGECTPTGEYELCESMASYDKN       115
JAP77260.1          EMNRTLQDETPCYAIPPHEALQMKLRAPRSCPLGLCENGVCKPTGKYEICESIATIKKM       114
XP_037290168.1      ETNRTLQDNTPCYVIPPQEALRMKLGAPRRCLLGLCENGACKPTGKYELCESIASYDQM       114
```

(b)

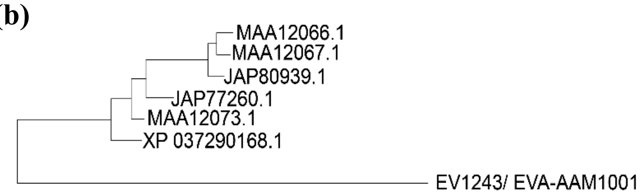

Supplementary Figure 3. Sequences of C_{10} evasins from *Rhipicephalus* species. (a) Multiple sequence alignments of a fourth subfamily of class A evasin-like proteins, all encoded by tick species from the genus *Rhipicephalus* and all containing the eight conserved cysteine residue from class A1 evasins (highlighted in yellow) as well as two additional cysteine residues (highlighted red) in positions distinct from class A3 evasins. Sequence names are the primary accession codes found in UniProt. Abbreviations used for tick species are *RAP, R. appendiculatus; RZA, R. zambeziensi and RMI, R. microplus.* (b) The unrooted tree (ClustalO, IQ-TREE) showing a separate clade of evasin-like sequences from *Rhipicephalus* species.

(a)

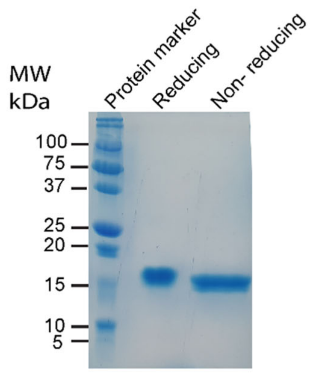

(b)

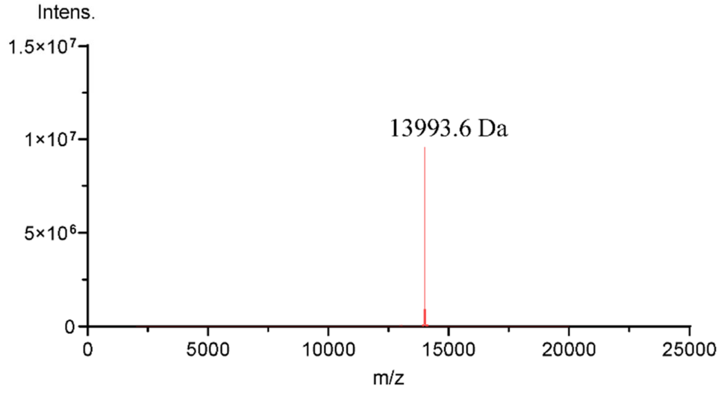

Supplementary Figure 4. Quality Control Analysis of purified EVA-A. (a) SDS-PAGE of wild type EVA-A under reducing and non-reducing conditions and stained with colloidal Coomassie blue. (b) Purified EVA-A (with a C-terminal linker-AVI tag; sequence ($(GGGGS)_3$ GLNDIFEAQKIEWHE) was analysed by liquid chromatography-electrospray ionisation mass spectrometry (LC MS-ESI). The expected mass of EVA-A with five disulfide bonds is 13994.42 Da.

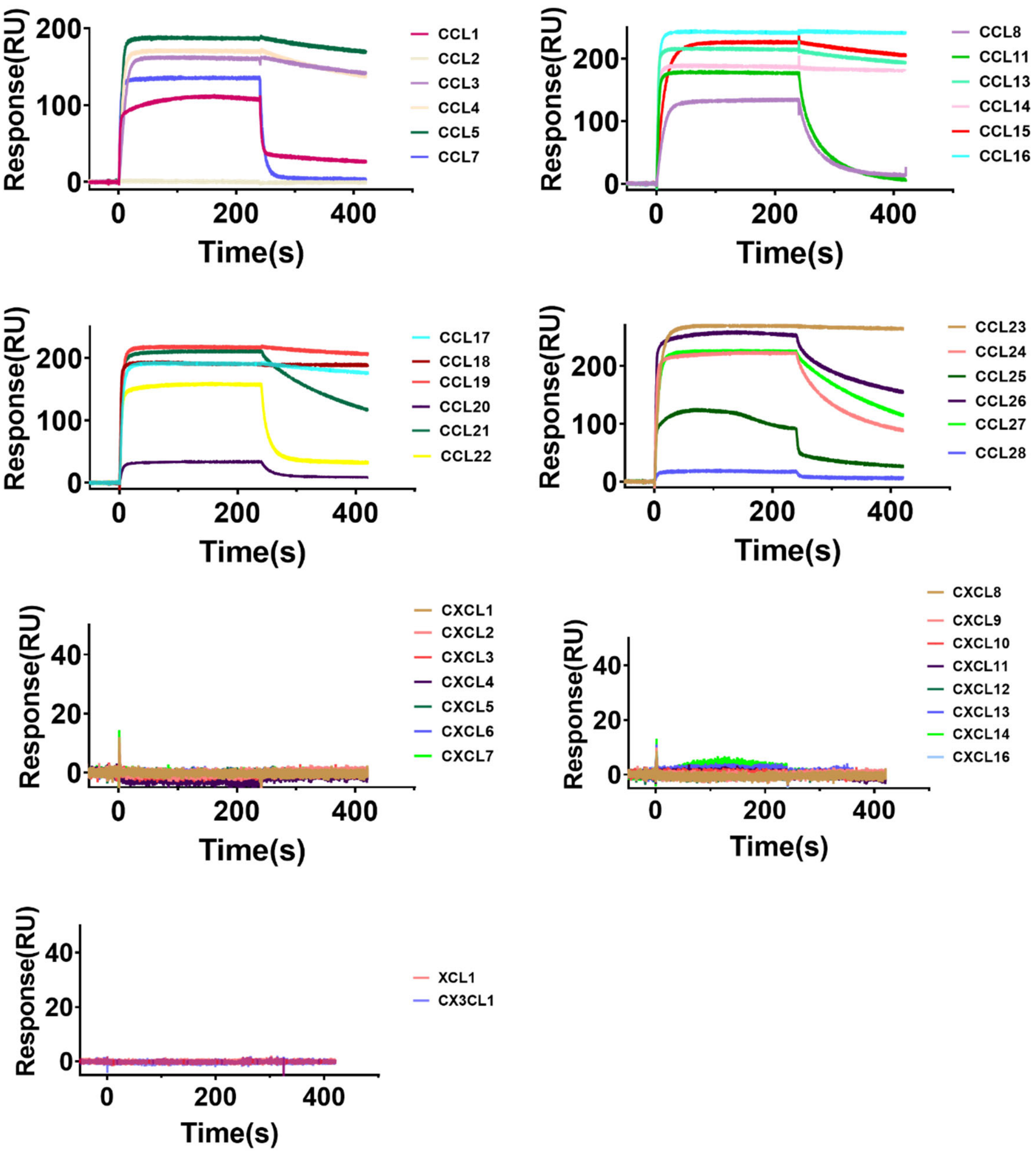

Supplementary Figure 5. EVA-A selectively binds to multiple human CC chemokines. Screening of EVA-A with multiple human chemokines (500 nM) by SPR using single-cycle kinetics at a single concentration. EVA-A selectively binds to CC chemokines but not to other classes of chemokines.

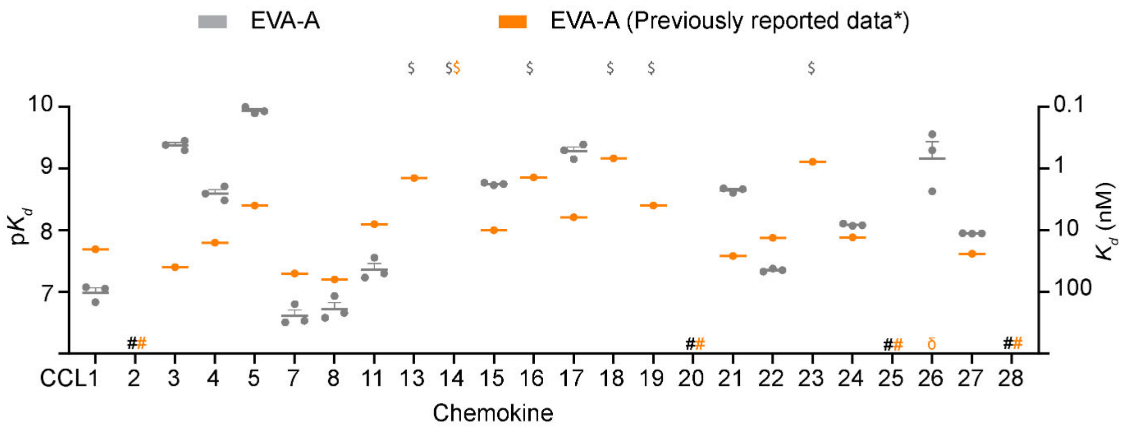

Supplementary Figure 6. Comparison of our EVA-A binding data with previously reported data. \$, $K_d < 0.1$ nM; #, no measurable binding at 500 nM chemokine concentration; δ, data unavailable. * Alenazi, Y. et al. Sci. Rep. 8, 6333 (2018) Statistical comparison is not possible because only a single K_d value was reported previously.

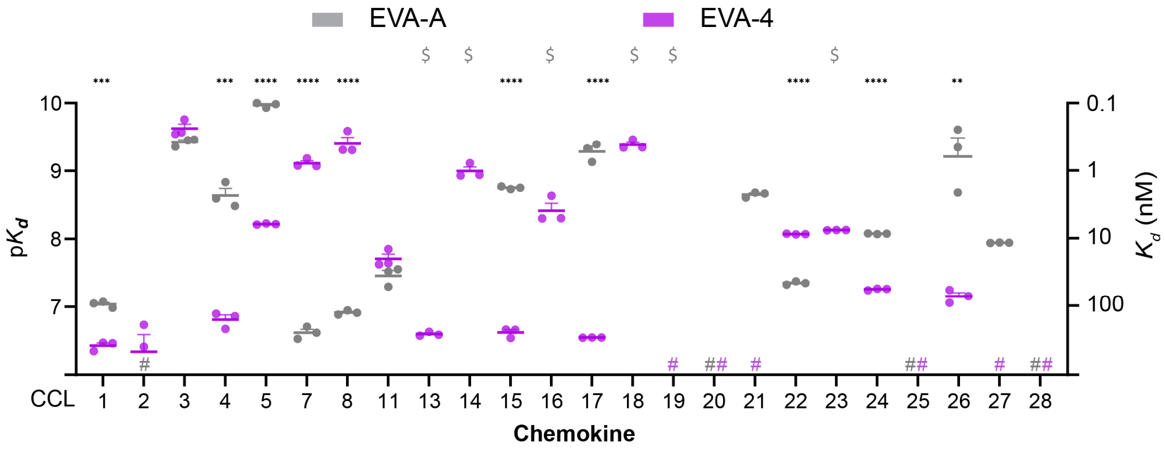

Supplementary Figure 7. Comparison of binding affinities of EVA-A and EVA-4. pK_d values for EVA-4 (expressed in human embryonic kidney cells) are from Aryal, P. et al. *J. Biol. Chem*. 298 (2022) DOI: https://doi.org/10.1016/j.jbc.2022.102382. Data are presented as mean ± SEM from three independent experiments. $, K_d < 0.1 nM; #, no measurable binding at 500 nM chemokine concentration; *p<0.05, **p<0.01, ***p<0.001, ****p<0.0001 (two-tailed t test with Holm- Šídák correction for multiple comparisons).

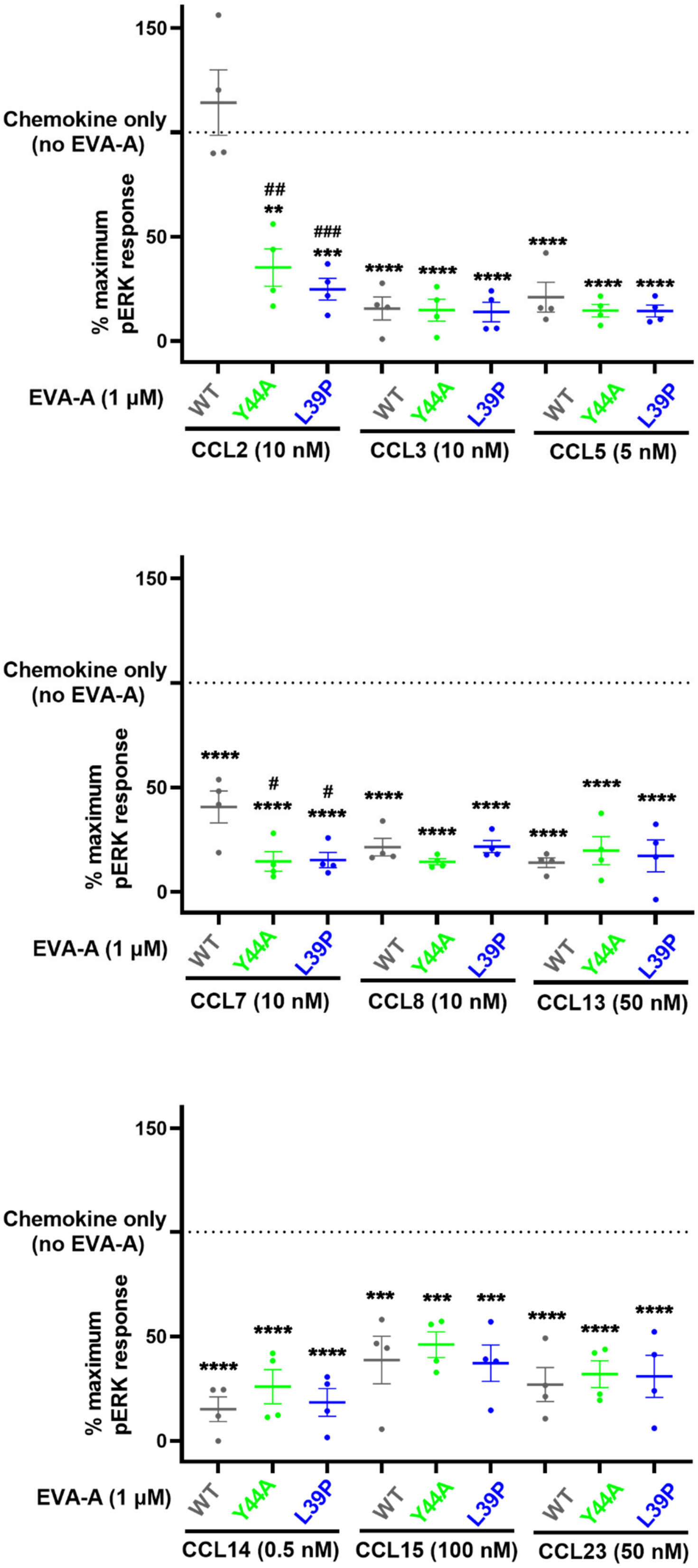

150
Chemokine only
(no EVA-A)
50
0
% maximum
pERK response
##
**
###
***
****
EVA-A (1 µM)
WT
Y44A
L39P
CCL2 (10 nM)
CCL3 (10 nM)
CCL5 (5 nM)
#
CCL7 (10 nM)
CCL8 (10 nM)
CCL13 (50 nM)
CCL14 (0.5 nM)
CCL15 (100 nM)
CCL23 (50 nM)

Supplementary Figure 8. EVA-A, EVA-A(L39P) and EVA-A(Y44A) inhibit the ability of chemokines to stimulate receptor mediated phosphorylation of extracellular signal-regulated kinase 1/2 (ERK). THP-1 monocytes that endogenously express the chemokine receptors CCR1 and CCR2 were treated with the chemokines (ligands of CCR1 and/or CCR2, at the indicated concentrations) or treated with mixtures of the chemokines (at the indicated concentrations) and evasins (at 1 μM concentration). The observed level of ERK phosphorylation (pERK response) was normalised to the response for each chemokine alone. Data points represent the average ± SEM of four independent experiments, each conducted in duplicate. *p<0.05, **p<0.01, ***p<0.001 and ****p<0.0001 relative to chemokine signals (one-way ANOVA with Dunnett's multiple comparisons test). #p<0.05, ##p<0.01, ### p<0.001 relative to chemokine signals inhibited by wild type EVA-A (one-way ANOVA with Dunnett's multiple comparisons test).

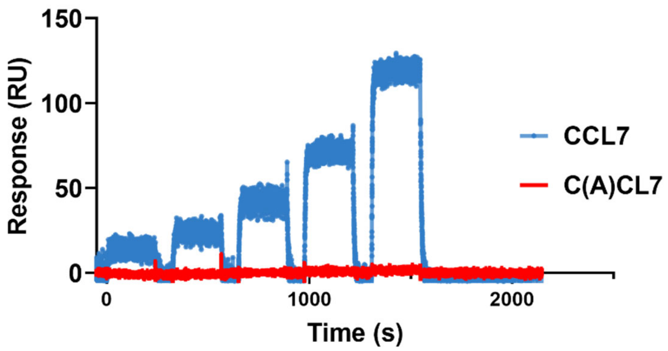

Supplementary Figure 9. SPR sensorgrams for CCL7 and C(A)CL7. Single-cycle kinetics SPR sensorgrams for CCL7 (sky blue) and C(A)CL7 (red) binding to EVA-A.

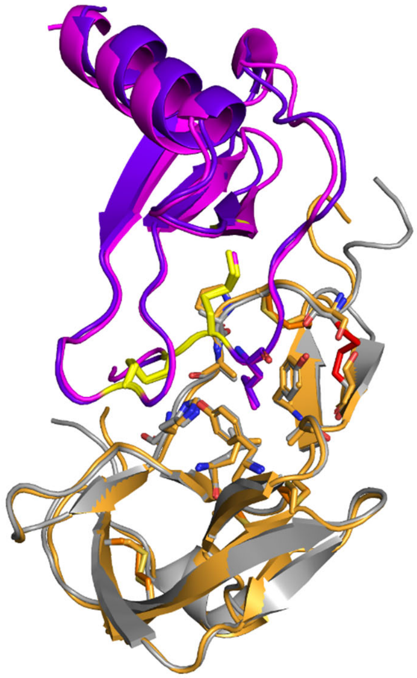

Supplementary Figure 10. Superimposition of EVA-A CCL17 and EVA-A(C_8) CCL17. Overlay of EVA-A and EVA-A(C_8) CCL17 shows the same fold despite the removal of an additional disulfide bond.

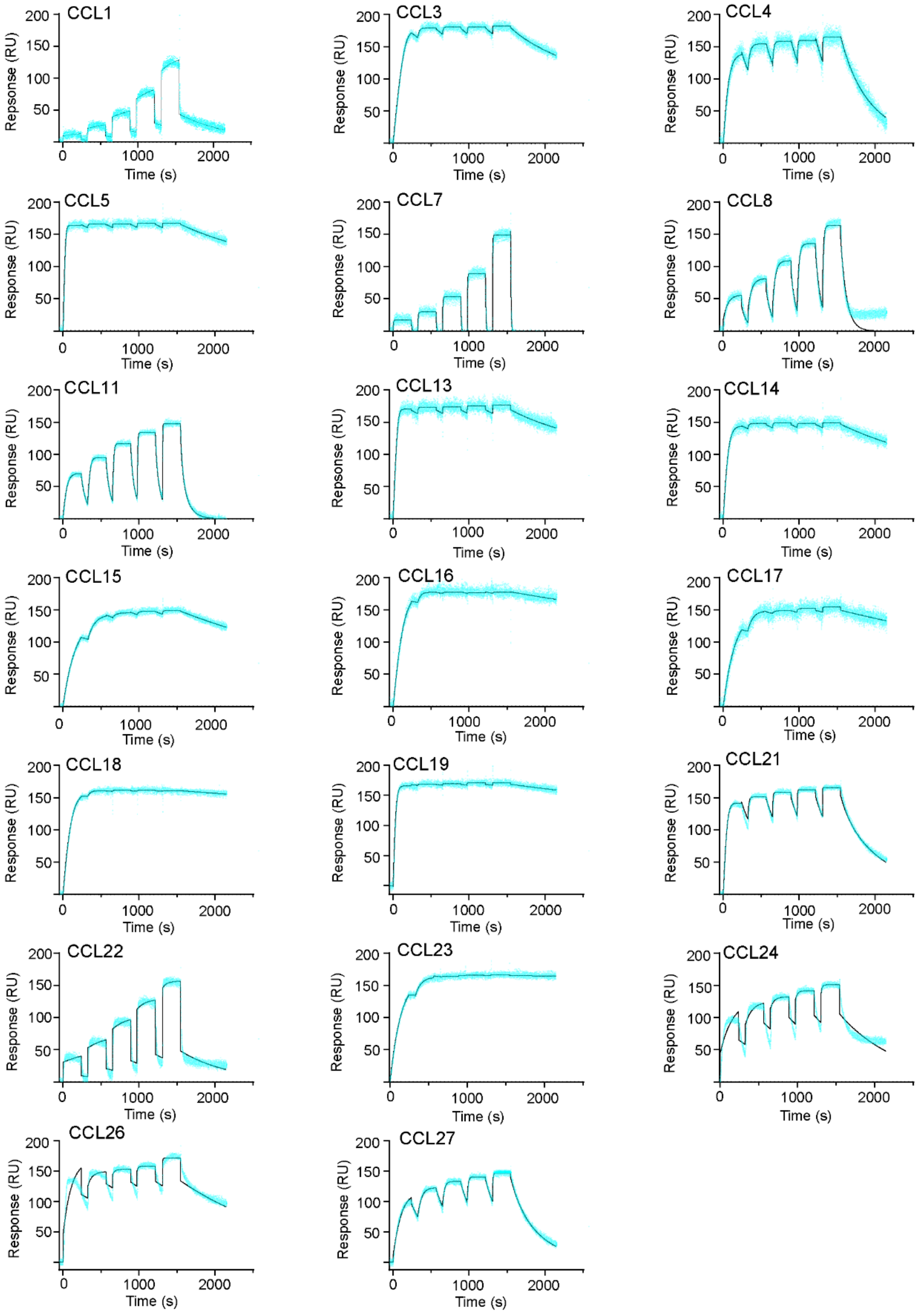

Supplementary Figure 11. Representative surface plasmon resonance (SPR) sensorgrams showing the binding of wild type EVA-A to CC chemokines, with experimental data in cyan and fitted curves in black.

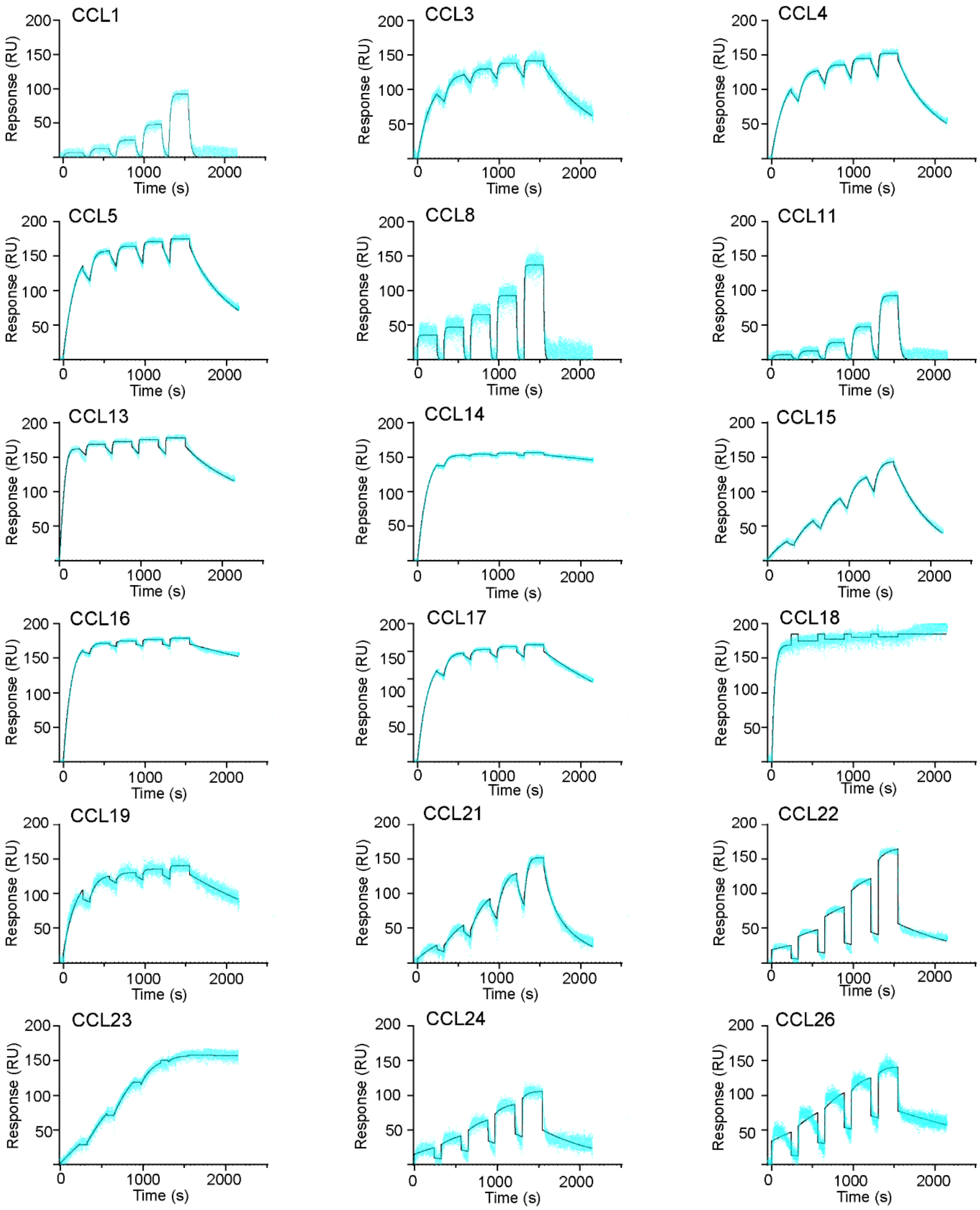

Supplementary Figure 12. Representative surface plasmon resonance (SPR) sensorgrams showing the binding of EVA-A(21-102) to CC chemokines, with experimental data in cyan and fitted curves in black.

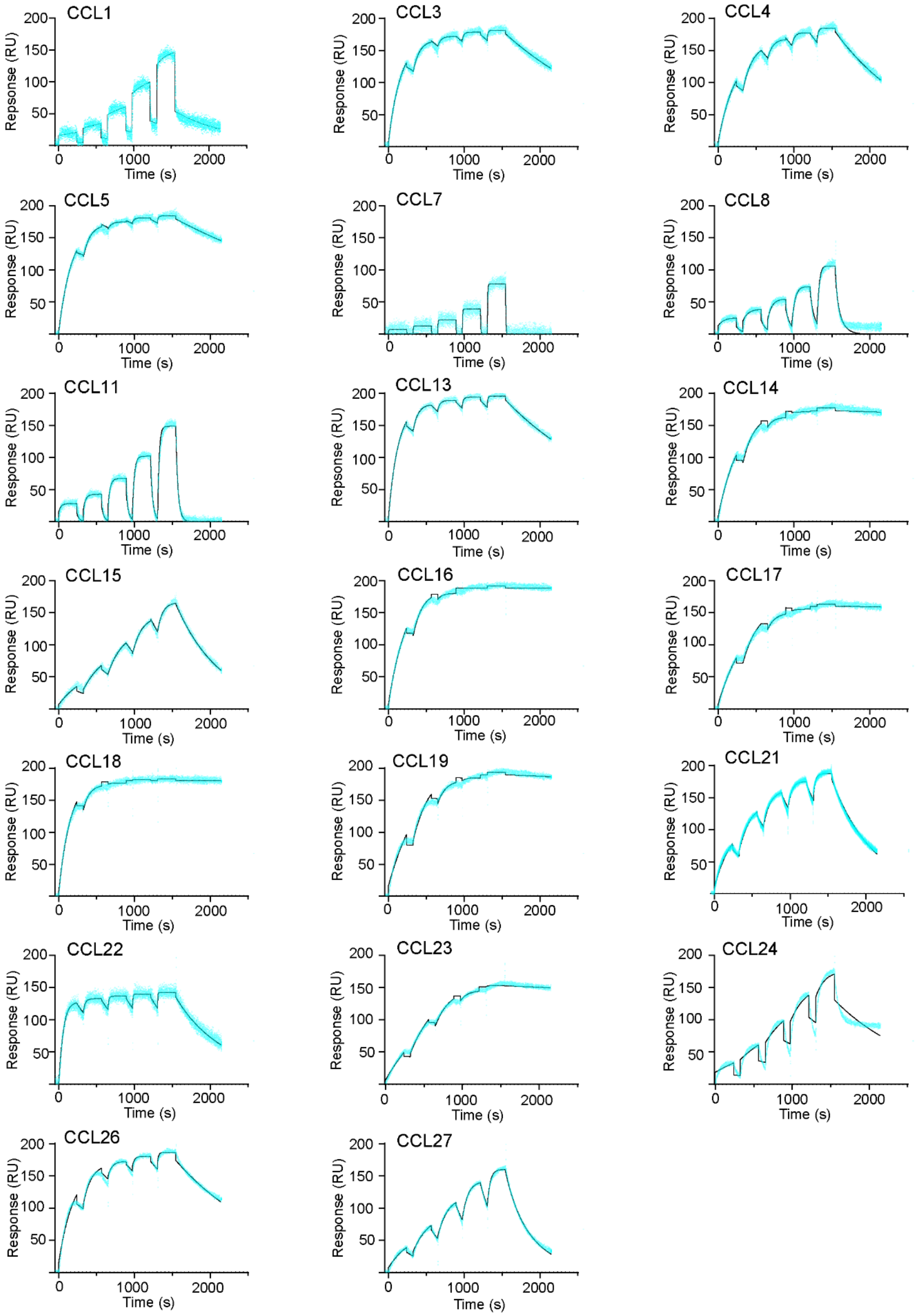

Supplementary Figure 13. Representative surface plasmon resonance (SPR) sensorgrams showing the binding EVA-A(1-98) to CC chemokines, with experimental data in cyan and fitted curves in black.

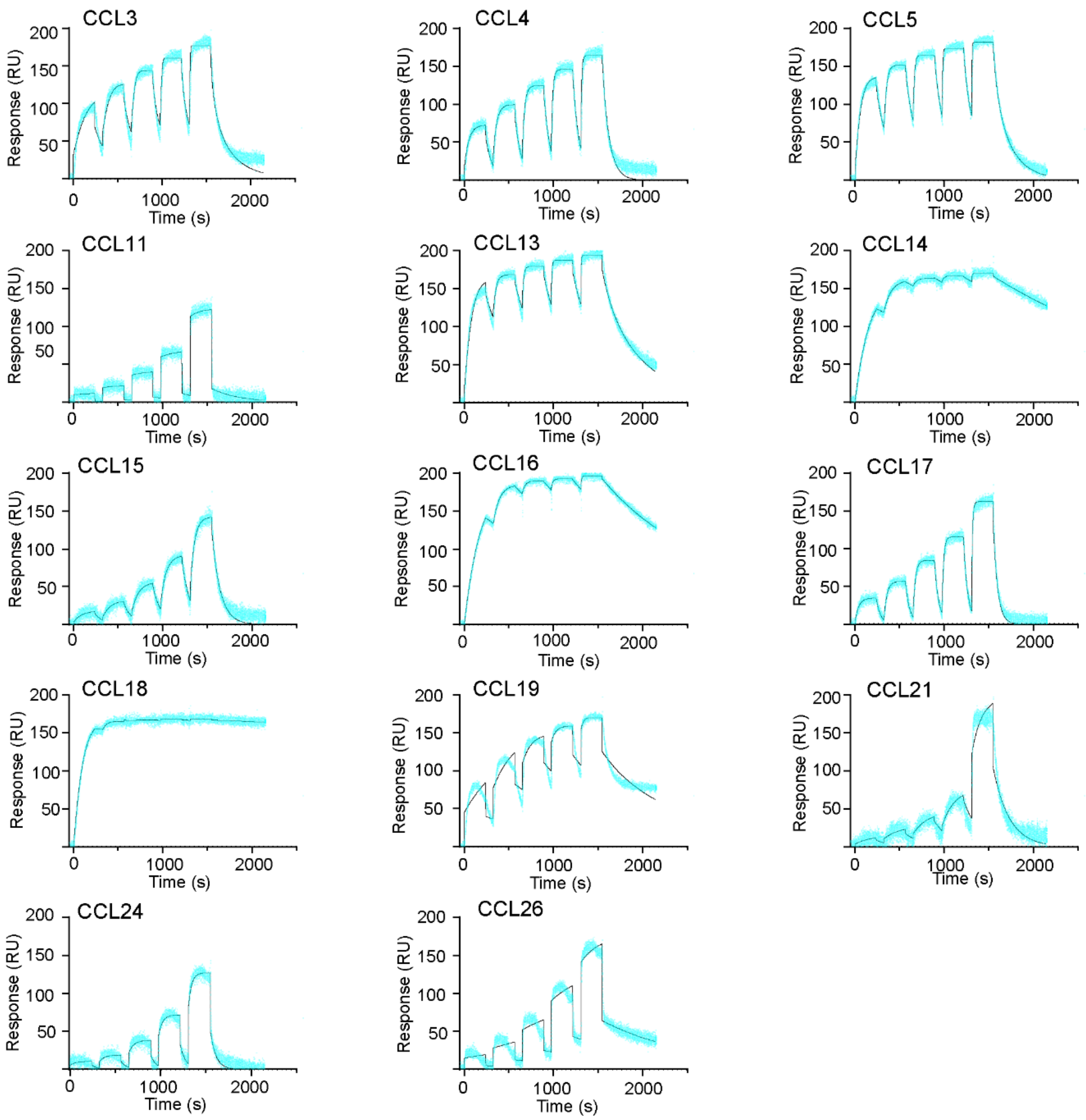

Supplementary Figure 14. Representative surface plasmon resonance (SPR) sensorgrams showing the binding of EVA-A(C_8) to CC chemokines, with experimental data in cyan and fitted curves in black.

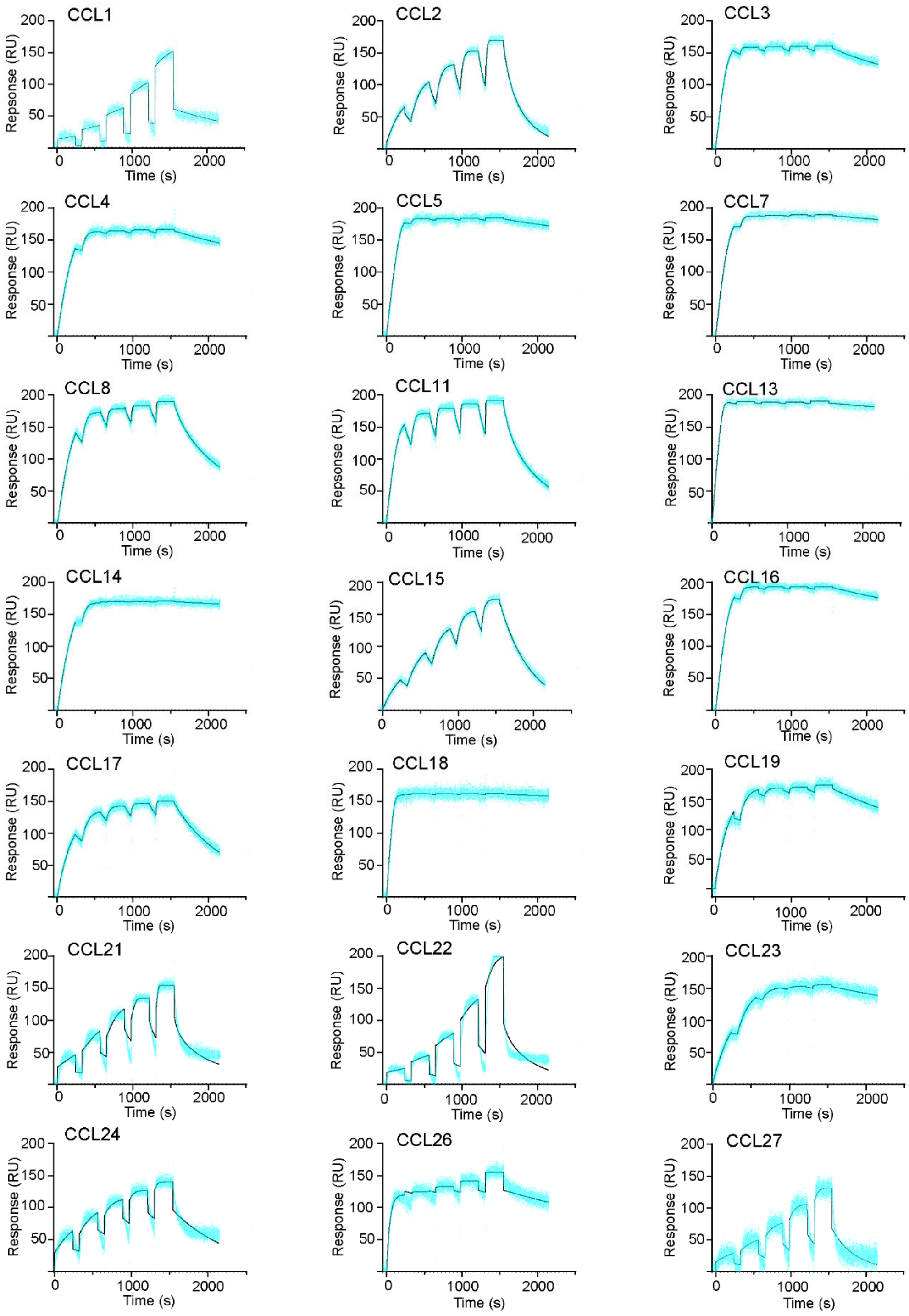

Supplementary Figure 15. Representative surface plasmon resonance (SPR) sensorgrams showing the binding of EVA-A(L39P) to CC chemokines, with experimental data in cyan and fitted curves in black.

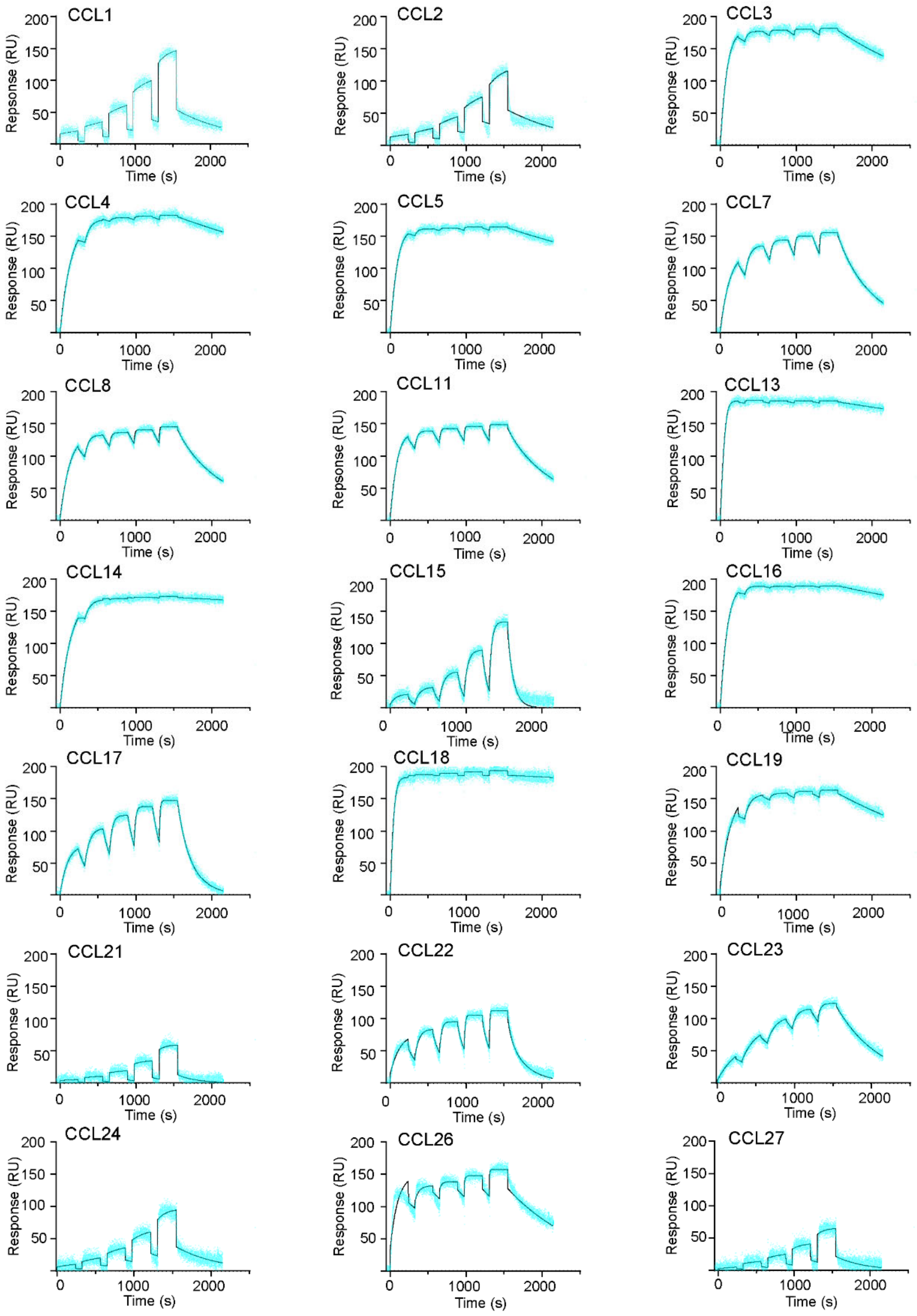

Supplementary Figure 16. Representative surface plasmon resonance (SPR) sensorgrams showing the binding of EVA-A(Y44A) to CC chemokines, with experimental data in cyan and fitted curves in black.

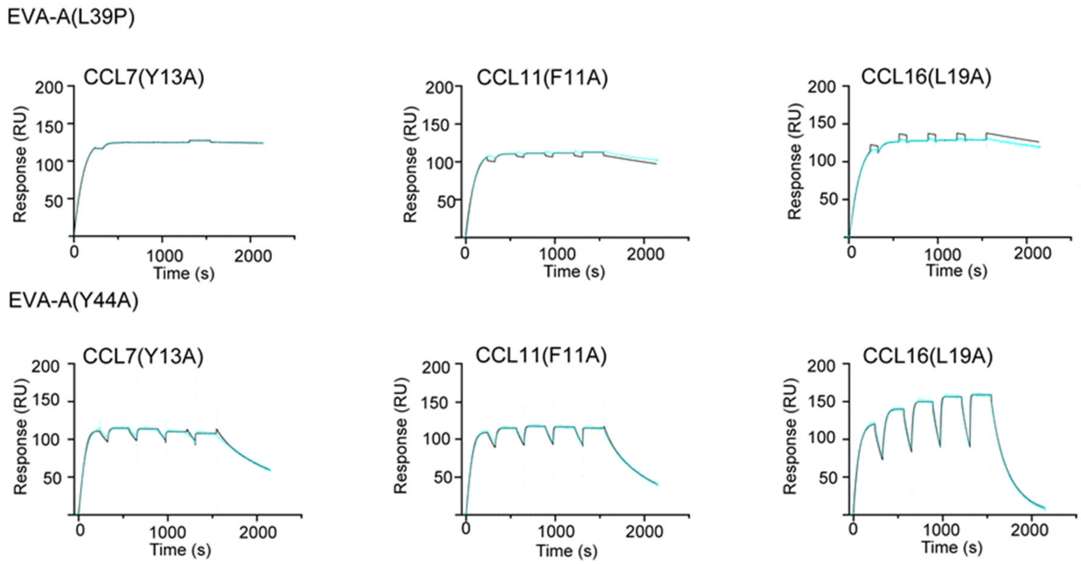

Supplementary Figure 17. Representative surface plasmon resonance (SPR) sensorgrams showing the binding of EVA-A mutants to the chemokine (CCL7, CCL11 and CCL16) variants prepared by substitution of CC+1 residue by alanine, with experimental data in cyan and fitted curves in black.

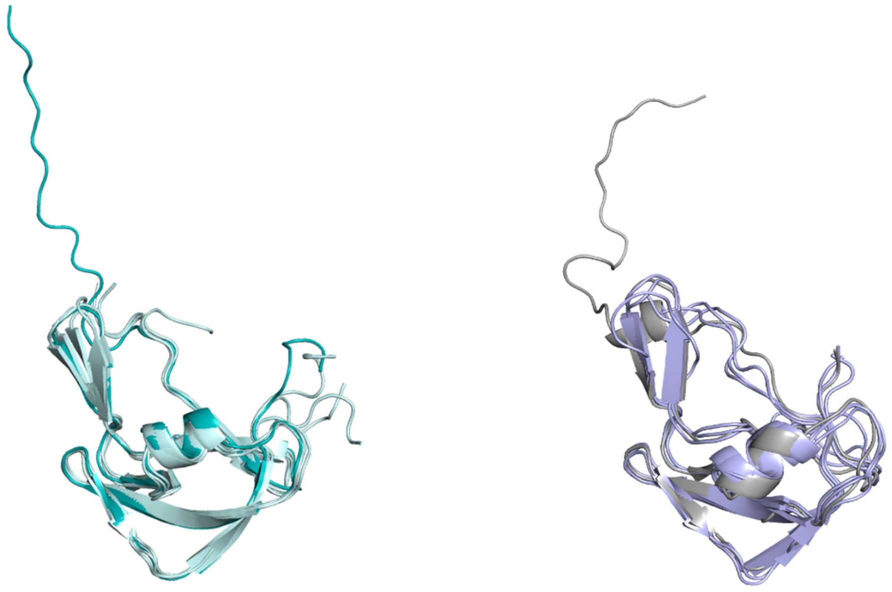

EVA-P overlaid with simulated structures

EVA-A overlaid with simulated structures

Supplementary Figure 18. Superimposition of the representative conformations of EVA-A and EVA-P from each of the three replicates MD simulations. (a) Figure showing the overlay of WT EVA-P (green teal) structure with three MD representative structures of EVA-P (pale cyan). (b) Figure showing the overlay of WT EVA-A (grey) structure with three MD representative structures of EVA-A (light blue). Both EVA-P and EVA-A retain the same fold over the MD simulations.

Residue no:	*22*	*25*	*39*	*44*	*51*	*57*
A0A0C9S461 (AAM)	C	P	L	Y	C	Y
A0A6M2E2L8 (ATL)	C	P	A	Y	C	Y
A0A6M2E4M9 (ATL)	C	L	V	Y	C	Y
A0A6M2E3I6 (ATL)	C	P	V	Y	C	Y
A0A6M2E5Z3 (ATL)	C	P	L	Y	C	Y
A0A6M2E374 (ATL)	C	P	L	Y	C	Y
A0A6M2E879 (ATL)	C	P	P	Y	C	E
A0A6M2E5F6 (ATL)	C	P	P	Y	C	E
A0A6M2E2U0 (ATL)	C	P	P	Y	C	E
A0A023G2M2 (ATT)	C	S	T	Q	C	T
A0A023G2G7 (ATT)	C	S	A	L	C	T
A0A023FT45 (APA)	C	P	P	Y	C	T
A0A1E1X165 (AAL)	C	P	Q	Y	C	T
A0A023FFD0 (ACA)	C	P	P	Y	C	Y

Supplementary Figure 19. Residues forming the CC+1 binding pocket in class A3 evasins.
Residues numbers are for EVA-A. Residues conserved with EVA-A are highlighted in pink.

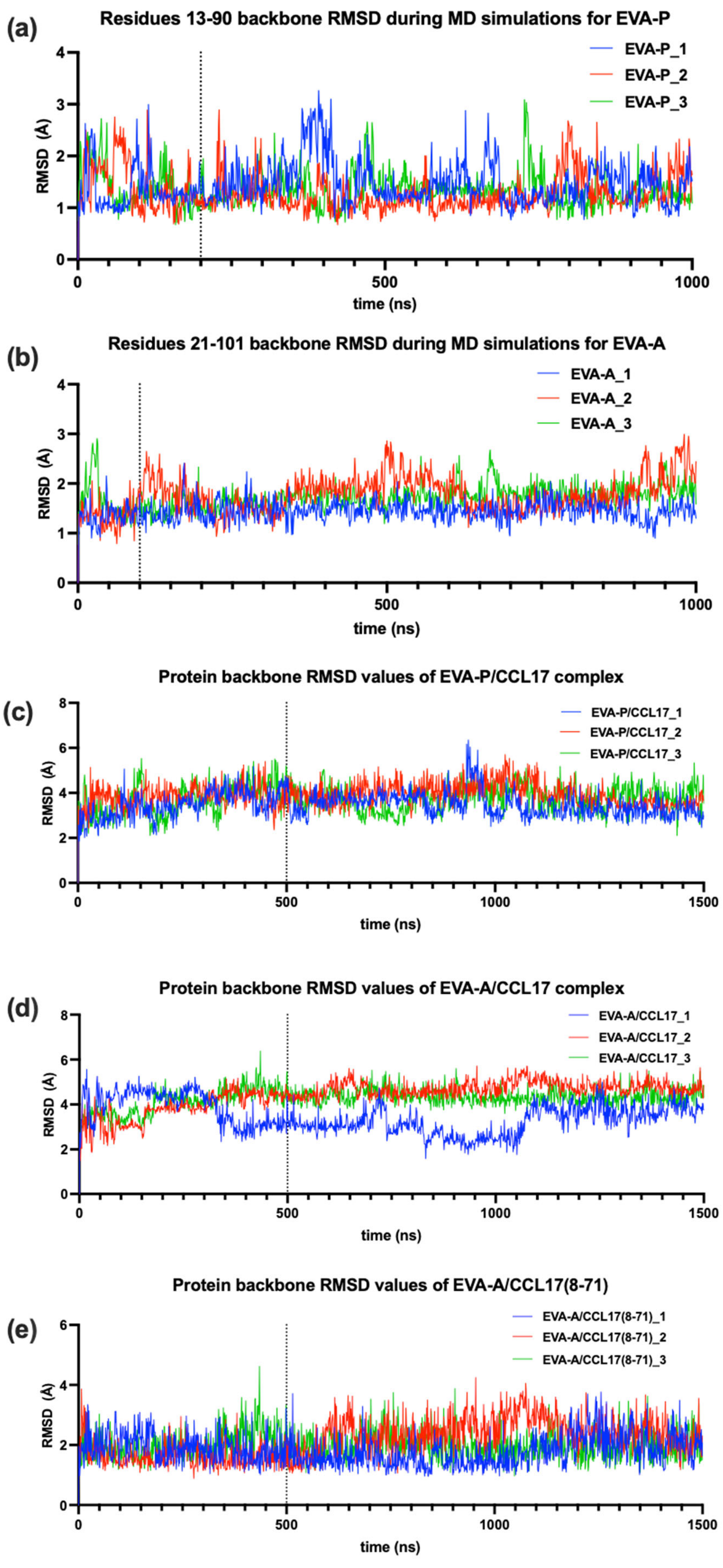

(a)
Residues 13-90 backbone RMSD during MD simulations for EVA-P
EVA-P_1
EVA-P_2
EVA-P_3
RMSD (Å)
0
1
2
3
4
0
500
1000
time (ns)
(b)
Residues 21-101 backbone RMSD during MD simulations for EVA-A
EVA-A_1
EVA-A_2
EVA-A_3
RMSD (Å)
0
1
2
3
4
0
500
1000
time (ns)
Protein backbone RMSD values of EVA-P/CCL17 complex
(c)
EVA-P/CCL17_1
EVA-P/CCL17_2
EVA-P/CCL17_3
RMSD (Å)
0
2
4
6
8
0
500
1000
1500
time (ns)
Protein backbone RMSD values of EVA-A/CCL17 complex
(d)
EVA-A/CCL17_1
EVA-A/CCL17_2
EVA-A/CCL17_3
RMSD (Å)
0
2
4
6
8
0
500
1000
1500
time (ns)
Protein backbone RMSD values of EVA-A/CCL17(8-71)
(e)
EVA-A/CCL17(8-71)_1
EVA-A/CCL17(8-71)_2
EVA-A/CCL17(8-71)_3
RMSD (Å)
0
2
4
6
0
500
1000
1500
time (ns)

Supplementary Figure 20. Protein backbone RMSD values during MD simulations. (a) Protein backbone RMSD values of residues 13-90 during MD simulations for N-terminal truncated EVA-P. Each simulation was conducted for 1 μs. All three replicates appeared to be equilibrated after 200 ns, thus simulation periods 200 ns – 1 μs were analysed. Total time for analysis was 800 ns x 3 = 2.4 μs. (b) Protein backbone RMSD values of residues 21-101 during MD simulations for N-terminal truncated EVA-A. Each simulation was conducted for 1 μs. All three replicates appeared to be equilibrated after 100 ns, thus simulation periods 100 ns – 1 μs were analysed. Total time for analysis was 900 ns x 3 = 2.7 μs. (c) Protein backbone RMSD values of EVA-P/CCL17 complex during MD simulations. The trajectories were first aligned by backbone atoms of EVA-P and then the overall protein (including both EVA-P and CCL17) RMSD values were calculated. For EVA-P/CCL17 complex, the simulations appeared to have equilibrated after 500 ns of each simulation, therefore, time periods 500 ns – 1.5 μs were analysed. Total time for analysis was 3 μs. (d) Protein backbone RMSD values of EVA-A/CCL17 complex during MD simulations. The trajectories were first aligned by backbone atoms of EVA-A and then the overall protein (including both EVA-P and CCL17) RMSD values were calculated. The overall protein backbone RMSD values appeared to have equilibrated after 350 ns in both simulations 2 and 3, but simulation 1 showed large fluctuations. Examination of the trajectories in simulation 1 showed the fluctuation may be due to movements of the flexible N-terminal region of CCL17 (residues 1-8). To confirm this, protein RMSD values including all backbone of EVA-A and backbone of residues 8 to 71 of CCL17 are plotted in (e). (e) Protein backbone RMSD values of EVA-A/CCL17(residue 8-71) complex during MD simulations. When the flexible N-terminal region has been removed from the RMSD calculation, it is clear simulation 1 has also equilibrated. Therefore, trajectories between 500 ns – 1.5 μs from each simulation were used for analysis. The total time for analysis was 3 μs.

Supplementary Table 1. Kinetic and equilibrium parameters for EVA-A binding to chemokines: fitted values of the association rate constant (k_a) and dissociation rate constant (k_d), the equilibrium dissociation constants (K_d) and negative logarithm of the equilibrium dissociation constant K_d in M (pK_d), presented as mean ± SEM from three independent SPR experiments. The representative K_d value listed is calculated from the mean value of pK_d.

**Chemokine**	$k_a \times 10^5$ [M^{-1}s^{-1}]	$k_d \times 10^{-3}$ [s^{-1}]	**pK_d**	K_d **[nM]**
CCL1	0.16 ± 0.03	1.55 ± 0.07	6.99 ± 0.08	107
CCL2	#	#	#	#
CCL3	14.8 ± 1.1	0.63 ± 0.09	9.38 ± 0.04	0.42
CCL4	4.40 ± 0.15	1.13 ± 0.15	8.60 ± 0.07	2.59
CCL5	38.7 ± 14.4	0.046 ± 0.171	9.943± 0.003	0.11
CCL7	2.03 ± 1.67	57.3 ± 50.8	6.61 ± 0.10	256
CCL8	3.17 ± 0.17	64.9 ± 16.6	6.72 ± 0.11	200
CCL11	11.6 ± 3.4	57.3 ± 25. 9	7.36 ± 0.10	45.6
CCL13	51.4 ± 5.7	$	$	$
CCL14	12.8 ± 1.5	$	$	$
CCL15	1.76 ± 0.03	0.31 ± 0.00	8.75 ± 0.01	1.77
CCL16	10.6 ± 2.0	$	$	$
CCL17	4.52 ± 0.81	0.23 ± 0.03	9.28 ± 0.07	0.54
CCL18	19.6 ± 10.9	$	$	$
CCL19	15.8 ± 0.4	$	$	$
CCL20	#	#	#	#
CCL21	12.4 ± 1.3	2.73 ± 0.14	8.65 ± 0.02	2.23
CCL22	0.33 ± 0.003	0.04	7.35 ± 0.01	44.6
CCL23	2.29 ± 0.01	$	$	$
CCL24	1.82 ± 0.19	1.48 ± 0.12	8.09 ± 0.01	8.19
CCL25	#	#	#	#
CCL26	19.4 ± 12.4	0.81 ± 0.19	9.16 ± 0.28	1.04
CCL27	3.18 ± 0.03	3.58 ± 0.03	7.95 ± 0.00	11.3
CCL28	#	#	#	#

$, ($K_d$ < 0.1 nM, pK_d > 10, $k_d < 10^{-4}$ s^{-1})

#, no measurable binding at 500 nM chemokine concentration

Supplementary Table 2. Kinetic and equilibrium parameters for EVA-A (21-102) binding to chemokines: fitted values of the association rate constant (k_a) and dissociation rate constant (k_d), the equilibrium dissociation constants (K_d) and negative logarithm of the equilibrium dissociation constant K_d in M (pK_d), presented as mean ± SEM from three independent SPR experiments. The representative K_d value listed is calculated from the mean value of pK_d.

**Chemokines**	$k_a \times 10^5$ **[M⁻¹s ⁻¹]**	$k_d \times 10^{-3}$ **[s⁻¹]**	**pK_d**	K_d ***[nM]***
CCL1	2.68 ± 2.54	180.35 ± 175.82	6.36 ± 0.10	457
CCL2	#	#	#	#
CCL3	4.49 ± 0.29	1.44 ± 0.01	8.49 ± 0.03	3.23
CCL4	4.61 ± 0.67	2.18 ± 0.06	8.31 ± 0.06	4.95
CCL5	7.77 ± 0.50	1.95 ± 0.07	8.60 ± 0.02	2.52
CCL7	#	#	#	#
CCL8	1.12 ± 0.97	34.39 ± 33.16	6.93 ± 0.29	169
CCL11	4.54 ± 0.53	218.17 ± 23.34	6.32 ± 0.10	507
CCL13	29.40 ± 0.99	0.87 ± 0.03	9.53 ± 0.01	0.30
CCL14	7.03 ± 0.06	0.08 ± 0.00	9.95 ± 0.01	0.11
CCL15	0.63 ± 0.02	2.14 ± 0.03	7.47 ± 0.01	34.0
CCL16	8.31 ± 0.14	0.19 ± 0.00	9.65 ± 0.00	0.22
CCL17	5.20 ± 0.10	0.54 ± 0.01	8.98 ± 0.01	1.04
CCL18	15.87 ± 3.35	$	$	$
CCL19	3.42 ± 0.21	0.53 ± 0.03	8.81 ± 0.05	1.58
CCL20	#	#	#	#
CCL21	2.24 ± 0.23	10.57 ± 1.01	7.33 ± 0.04	47.53
CCL22	0.26 ± 0.02	1.61 ± 0.19	7.22 ± 0.06	61.60
CCL23	0.87 ± 0.32	0.04 ± 0.02	9.40 ± 0.22	0.51
CCL24	0.46 ± 0.09	0.91 ± 0.20	7.70 ± 0.01	19.93
CCL25	#	#	#	#
CCL26	0.53 ± 0.06	0.49 ± 0.05	8.03 ± 0.02	9.24
CCL27	#	#	#	#
CCL28	#	#	#	#

$, ($K_d$ < 0.1 nM, pK_d > 10, $k_d < 10^{-4}$ s⁻¹)

#, no measurable binding at 500 nM chemokine concentration

Supplementary Table 3. Kinetic and equilibrium parameters for EVA-A(1-98) binding to chemokines: fitted values of the association rate constant (k_a) and dissociation rate constant (k_d), the equilibrium dissociation constants (K_d) and negative logarithm of the equilibrium dissociation constant K_d in M (pK_d), presented as mean ± SEM from three independent SPR experiments. The representative K_d value listed is calculated from the mean value of pK_d.

**Chemokines**	$k_a \times 10^5$ [M^{-1}s^{-1}]	$k_d \times 10^{-3}$ [s^{-1}]	pK_d	K_d [nM]
CCL1	0.06 ± 0.02	1.68 ± 2.55	6.43 ± 0.13	406
CCL2	#	#	#	#
CCL3	3.45 ± 0.11	0.58 ± 0.64	8.75 ± 0.02	1.77
CCL4	1.65 ± 0.56	0.87 ± 0.94	8.18 ± 0.21	8.56
CCL5	3.54 ± 0.10	0.33 ± 0.41	8.99 ± 0.02	1.03
CCL7	0.31 ± 0.26	1.79 ± 0.51	6.27 ± 0.34	1014
CCL8	1.73 ± 1.35	34.43 ± 25.29	6.82 ± 0.12	163
CCL11	1.01 ± 0.14	53.65 ± 34.54	6.31 ± 0.01	487
CCL13	4.58 ± 0.05	0.63 ± 0.67	8.85 ± 0.01	1.42
CCL14	2.39 ± 0.29	$	$	$
CCL15	0.53 ± 0.00	1.62 ± 1.73	7.50 ± 0.01	31.67
CCL16	2.58 ± 0.04	$	$	$
CCL17	1.59 ± 0.03	$	$	$
CCL18	4.43 ± 0.27	$	$	$
CCL19	1.44 ± 0.05	0.03 ± 0.01	9.82 ± 0.12	0.16
CCL20	#	#	#	#
CCL21	1.26 ± 0.24	2.33 ± 1.74	7.79 ± 0.14	17.8
CCL22	0.29 ± 0.08	0.84 ± 0.37	6.90 ± 0.00	125
CCL23	1.44 ± 0.33	0.02 ± 0.04	9.48 ± 0.26	0.45
CCL24	0.36 ± 0.06	1.11 ± 0.89	7.55 ± 0.04	28.10
CCL25	#	#	#	#
CCL26	3.57 ± 0.39	0.48 ± 0.67	8.75 ± 0.10	1.89
CCL27	0.95 ± 0.13	4.09 ± 3.67	7.39 ± 0.06	41.73
CCL28	#	#	#	#

$, ($K_d < 0.1$ nM, p$K_d > 10$, $k_d < 10^{-4}$ s^{-1})

#, no measurable binding at 500 nM chemokine concentration

Supplementary Table 4. Kinetic and equilibrium parameters for EVA-A(C_8) binding to chemokines: fitted values of the association rate constant (k_a) and dissociation rate constant (k_d), the equilibrium dissociation constants (K_d) and negative logarithm of the equilibrium dissociation constant K_d in M (pK_d), presented as mean ± SEM from three independent SPR experiments. The representative K_d value listed is calculated from the mean value of pK_d.

**Chemokines**	$k_a \times 10^5$ [$M^{-1}s^{-1}$]	$k_d \times 10^{-3}$ [s^{-1}]	**pK_d**	K_d **[nM]**
CCL1	#	#	#	#
CCL2	#	#	#	#
CCL3	10.84 ± 8.78	46.38 ± 38.37	7.40 ± 0.02	40.3
CCL4	2.93 ± 0.98	21.54 ± 8.32	7.15 ± 0.02	71.3
CCL5	7.16 ± 0.12	11.49 ± 0.28	7.79 ± 0.00	16.0
CCL7	1.77 ± 0.53	166.19 ± 76.01	6.22 ± 0.24	765
CCL8	#	#	#	#-
CCL11	2.36 ± 1.22	195.99 ± 104.04	6.22 ± 0.14	663
CCL13	14.73 ± 1.87	4.23 ± 0.32	8.54 ± 0.03	2.91
CCL14	3.86 ± 0.56	0.48 ± 0.02	8.89 ± 0.06	1.30
CCL15	0.22 ± 0.01	12.75 ± 1.29	6.25 ± 0.05	577
CCL16	3.96 ± 0.55	0.75 ± 0.08	8.72 ± 0.01	1.90
CCL17	2.54 ± 0.63	21.69 ± 2.10	7.04 ± 0.10	97.1
CCL18	8.37 ± 0.55	$	$	$
CCL19	2.45 ± 1.60	3.45 ± 1.99	7.80 ± 0.05	16.1
CCL20	#	#	#	#
CCL21	#	#	#	#
CCL22	#	#	#	#
CCL23	0.74 ± 0.14	1.31 ± 0.13	7.74 ± 0.14	20.3
CCL24	0.18 ± 0.03	8.69 ± 4.01	6.39 ± 0.13	447
CCL25	#	#	#	#
CCL26	0.14 ± 0.02	0.99 ± 0.03	7.16 ± 0.05	70.9
CCL27	#	#	#	#
CCL28	#	#	#	#

$, ($K_d$ < 0.1 nM, pK_d > 10, $k_d < 10^{-4}$ s^{-1})

#, no measurable binding at 500 nM chemokine concentration

Supplementary Table 5. Kinetic and equilibrium parameters for EVA-A(L39P) binding to chemokines: fitted values of the association rate constant (k_a) and dissociation rate constant (k_d), the equilibrium dissociation constants (K_d) and negative logarithm of the equilibrium dissociation constant K_d in M (pK_d), presented as mean ± SEM from three independent SPR experiments. The representative K_d value listed is calculated from the mean value of pK_d.

**Chemokines**	**$k_a \times 10^5$ [$M^{-1}s^{-1}$]**	**$k_d \times 10^{-3}$ [s^{-1}]**	**pK_d**	**K_d [nM]**
CCL1	0.17 ± 0.02	1.67 ± 0.64	7.26 ± 0.08	57.5
CCL2	3.29 ± 0.16	35.98 ± 28.10	7.61 ± 0.04	24.9
CCL3	12.20 ± 2.87	0.40 ± 0.02	9.51 ± 0.09	0.32
CCL4	8.05 ± 0.48	0.23 ± 0.04	9.52 ± 0.02	0.30
CCL5	15.27 ± 2.49	$	$	$
CCL7	10.50 ± 1.27	$	$	$
CCL8	9.71 ± 0.58	2.18 ± 0.51	8.59 ± 0.04	2.59
CCL11	14.00 ± 0.96	3.72 ± 1.19	8.48 ± 0.03	3.32
CCL13	32.57 ± 4.61	$	$	$
CCL14	24.08 ± 17.06	0.13 ± 0.08	10.35 ± 0.03	0.04
CCL15	1.02 ± 0.03	4.67 ± 1.93	7.58 ± 0.03	26.6
CCL16	11.83 ± 0.17	0.16 ± 0.04	9.77 ± 0.04	0.17
CCL17	4.39 ± 0.31	4.05 ± 1.40	8.26 ± 0.23	7.49
CCL18	37.43 ± 3.21	$	$	$
CCL19	2.78 ± 0.36	0.36 ± 0.03	8.88 ± 0.06	1.34
CCL20	#	#	#	#
CCL21	1.97 ± 0.31	4.84 ± 1.01	7.62 ± 0.05	24.5
CCL22	10.31 ± 1.50	2.92 ± 1.34	8.84 ± 0.05	1.46
CCL23	1.40 ± 0.30	0.67 ± 0.50	8.96 ± 0.08	1.14
CCL24	1.11 ± 0.17	1.21 ± 0.10	8.02 ± 0.02	9.58
CCL25	#	#	#	#
CCL26	26.33 ± 6.06	0.78 ± 0.31	9.73 ± 0.01	0.19
CCL27	0.45 ± 0.06	3.09 ± 0.26	7.20 ± 0.06	63.9
CCL28	#	#	#	#

$, ($K_d$ < 0.1 nM, pK_d > 10, $k_d < 10^{-4}$ s^{-1})

#, no measurable binding at 500 nM chemokine concentration

Supplementary Table 6. Kinetic and equilibrium parameters for EVA-A(Y44A) binding to chemokines: fitted values of the association rate constant (k_a) and dissociation rate constant (k_d), the equilibrium dissociation constants (K_d) and negative logarithm of the equilibrium dissociation constant K_d in M (pK_d), presented as mean ± SEM from three independent SPR experiments. The representative K_d value listed is calculated from the mean value of pK_d.

**Chemokines**	$k_a \times 10^5$ [M^{-1}s^{-1}]	$k_d \times 10^{-3}$ [s^{-1}]	pK_d	K_d [nM]
CCL1	0.57 ± 0.20	2.56 ± 0.63	7.33 ± 0.10	49.9
CCL2	2.91 ± 2.76	31.99 ± 30.09	6.95 ± 0.13	123
CCL3	5.66 ± 2.03	0.44 ± 0.01	9.06 ± 0.14	0.95
CCL4	3.19 ± 0.72	0.20 ± 0.02	9.19 ± 0.05	0.66
CCL5	9.81 ± 1.88	0.21 ± 0.02	9.66 ± 0.09	0.23
CCL7	4.19 ± 0.55	1.99 ± 0.15	8.32 ± 0.03	4.81
CCL8	4.74 ± 1.33	1.56 ± 0.13	8.45 ± 0.08	3.62
CCL11	8.77 ± 0.96	1.50 ± 0.10	8.76 ± 0.02	1.73
CCL13	26.83 ± 4.64	$	$	$
CCL14	4.81 ± 0.19	0.05 ± 0.01	9.99 ± 0.08	0.11
CCL15	0.79 ± 0.13	11.74 ± 1.63	6.83 ± 0.13	161.30
CCL16	6.64 ± 1.21	0.13 ± 0.01	9.71 ± 0.08	0.20
CCL17	1.66 ± 1.10	5.10 ± 0.50	7.32 ± 0.25	61.9
CCL18	16.93 ± 2.24	$	$	$
CCL19	4.90 ± 0.40	0.36 ± 0.02	9.13 ± 0.05	0.75
CCL20	#	#	#	#
CCL21	0.36 ± 0.09	3.56 ± 0.68	7.00 ± 0.20	118.9
CCL22	7.66 ± 1.35	8.18 ± 1.87	7.98 ± 0.12	11.2
CCL23	1.00 ± 0.15	1.76 ± 0.05	7.74 ± 0.08	18.8
CCL24	0.26 ± 0.01	1.79 ± 0.26	7.18 ± 0.08	68.6
CCL25	#	#	#	#
CCL26	3.81 ± 1.12	1.01 ± 0.20	8.56 ± 0.11	2.94
CCL27	0.52 ± 0.10	6.39 ± 3.53	7.02 ± 0.15	109.7
CCL28	#	#	#	#

$, ($K_d$ < 0.1 nM, pK_d > 10, $k_d < 10^{-4}$ s^{-1})

#, no measurable binding at 500 nM chemokine concentration

Supplementary Table 7. Kinetic and equilibrium parameters for EVA-A binding to chemokines: fitted values of the association rate constant (k_a) and dissociation rate constant (k_d), the equilibrium dissociation constants (K_d) and negative logarithm of the equilibrium dissociation constant K_d in M (pK_d), presented as mean ± SEM from three independent SPR experiments. The representative K_d value listed is calculated from the mean value of pK_d.

Chemokines	$k_a \times 10^5$ [$M^{-1}s^{-1}$]	$k_d \times 10^{-3}$ [s^{-1}]	pK_d	K_d [nM]
CCL7(Y13A)	9.66 ± 2.97	$	$	$
CCL11(F11A)	10.99 ± 1.42	0.25 ± 0.003	9.63 ± 0.05	0.24
CCL16(L19A)	7.31 ± 0.95	0.20 ± 0.01	9.57 ± 0.06	0.27

$, ($K_d < 0.1$ nM, p$K_d > 10$, $k_d < 10^{-4}$ s^{-1})

Supplementary Table 8. Kinetic and equilibrium parameters for EVA-A(L39P) binding to chemokines: fitted values of the association rate constant (k_a) and dissociation rate constant (k_d), the equilibrium dissociation constants (K_d) and negative logarithm of the equilibrium dissociation constant K_d in M (pK_d), presented as mean ± SEM from three independent SPR experiments. The representative K_d value listed is calculated from the mean value of pK_d.

Chemokines	$k_a \times 10^5$ [$M^{-1}s^{-1}$]	$k_d \times 10^{-3}$ [s^{-1}]	pK_d	K_d [nM]
CCL7(Y13A)	6.85 ± 0.12	$	$	$
CCL11(F11A)	7.07 ± 0.99	0.35 ± 0.02	9.30 ± 0.04	0.51 \
CCL16(L19A)	5.60 ± 0.34	0.85 ± 0.05	8.82 ± 0.00	1.53 \

$, ($K_d < 0.1$ nM, p$K_d > 10$, $k_d < 10^{-4}$ s^{-1})

Supplementary Table 9. Kinetic and equilibrium parameters for EVA-A(Y44A) binding to chemokines: fitted values of the association rate constant (k_a) and dissociation rate constant (k_d), the equilibrium dissociation constants (K_d) and negative logarithm of the equilibrium dissociation constant K_d in M (pK_d), presented as mean ± SEM from three independent SPR experiments. The representative K_d value listed is calculated from the mean value of pK_d.

Chemokines	$k_a \times 10^5$ [$M^{-1}s^{-1}$]	$k_d \times 10^{-3}$ [s^{-1}]	pK_d	K_d [nM]
CCL7(Y13A)	4.41 ± 0.25	8.80 ± 0.24	7.70 ± 0.02	20.0
CCL11(F11A)	8.02 ± 0.47	2.26 ± 0.07	8.55 ± 0.02	2.83
CCL16(L19A)	1.07 ± 0.22	1.51 ± 0.11	7.83 ± 0.12	15.9

Supplementary Table 10. H-bond established during MD simulations of EVA-P:CCL17 and EVA-A:CCL17. High occupancy hydrogen bonds are in red.

EVA-P:CCL17			EVA-A:CCL17		
**Donor**	**Acceptor**	**Average**	**Donor**	**Acceptor**	**Average**
Cys50$^{CCL17}_{Main}$	Thr16$^{EVA-P}_{Main}$	39.96%	Cys50$^{CCL17}_{Main}$	Ala23$^{EVA-A}_{Main}$	61.77%
Phe19$^{EVA-P}_{Main}$	Cys10$^{CCL17}_{Main}$	62.94%	Ala26$^{EVA-A}_{Main}$	Cys10$^{CCL17}_{Main}$	36.03%
Cys10$^{CCL17}_{Main}$	Phe19$^{EVA-P}_{Main}$	35.03%	Cys10$^{CCL17}_{Main}$	Ala26$^{EVA-A}_{Main}$	17.42%
Val21$^{EVA-P}_{Main}$	Arg8$^{CCL17}_{Main}$	17.12%	His28$^{EVA-A}_{Main}$	Arg8$^{CCL17}_{Main}$	12.45%
Arg8$^{CCl17}_{Side}$	Gly92$^{EVA-P}_{Main}$	12.09%	Arg8$^{CCL17}_{Side}$	Asn30$^{EVA-A}_{Side}$	14.99%
Ala29$^{EVA-P}_{Main}$	Asp33$^{CCL17}_{Main}$	15.68%	Leu37$^{EVA-A}_{Main}$	Asp33$^{CCL17}_{Main}$	51.41%
Asn27$^{EVA-P}_{Side}$	Asp33$^{CCL17}_{Side}$	10.62%	Tyr44$^{EVA-A}_{Side}$	Glu13$^{CCL17}_{Side}$	15.72%

Supplementary Table 11. X-ray diffraction data collection and refinement statistics. Values in parentheses are for the outermost resolution shell.

	**EVA-A:CCL7**	**EVA-A:CCL11**	**EVA-A:CCL16**	**EVA-A:CCL17**
PDB ID	7SCU	7SCS	7SCT	7SCV
**Data collection**				
Wavelength (Å)	0.953	0.953	0.953	0.953
Resolution range	48.59 - 1.86 (1.90 - 1.86)	41.58 - 1.51 (1.54 - 1.51)	48.13 - 1.84 (1.88 - 1.84)	44.69 – 2.01 (2.07 – 2.01)
Space group	$P\ 4_1\ 2_1\ 2$	$P\ 4_1\ 2_1\ 2$	I 4 2 2	$P\ 3_1\ 2\ 1$
Cell dimensions				
a, b, c (Å)	71.2 71.2 66.4	75.0 75.0 66.9	96.2 96.2 84.7	88.0 88.0 55.1
a, b, g (°)	90 90 90	90 90 90	90 90 90	90 90 120
R_{merge}	0.102 (1.438)	0.075 (1.130)	0.076 (1.518)	0.114 (1.460)
R_{meas}	0.106 (1.497)	0.077 (1.339)	0.077 (1.549)	0.117 (1.502)
R_{pim}	0.021 (0.299)	0.015 (0.270)	0.015 (0.304)	0.026 (0.346)
I/s(I)	20.0 (2.7)	23.9 (2.9)	23.0 (2.7)	17.4 (2.4)
$CC_{1/2}$	0.996 (0.670)	1.000 (0.774)	0.999 (0.829)	0.999 (0.699)
Completeness (%)	99.8 (96.5)	99.8 (96.9)	99.9 (98.6)	99.7 (96.5)
Total reflections	377698 (21922)	776078 (33204)	459548 (26148)	330673 (21345)
Unique reflections	14877 (912)	30361 (1433)	17690 (1051)	16612 (1165)
Multiplicity	25.4 (24.0)	25.6 (23.2)	26.0 (24.9)	19.9 (18.3)
**Refinement**				
R_{work} / R_{free}	0.193 / 0.210	0.184 / 0.206	0.203 / 0.210	0.236 / 0.251
No. of atoms	1214	1331	1248	1177
Macromolecules	1124	1174	1169	1089
Solvent	90	157	79	88
Average B factors	38.3	29.6	50.3	50.4
Macromolecules	38.3	28.4	50.6	50.4
Solvent	38.3	38.6	46.0	50.8
r. m. s. deviations				
Bond lengths (Å)	0.003	0.009	0.007	0.002
Bond angles (°)	0.67	1.21	1.08	0.62
Ramachandran statistics (%)				
Favoured	99.31	97.97	97.28	96.53
Allowed	0.69	2.03	2.72	3.47
Outliers	0	0	0	0
Clashscore	1.38	6.44	2.18	4.78
MolProbity score	0.87	1.36	1.13	1.47

Supplementary Table 11. ***(continued)***

	**EVA-A (Y44A): CCL2**	**EVA-A (Y44A): CCL7**	**EVA-A (L39P): CCL7**	**EVA-A(C_8): CCL17**	**EVA-A: CCL7(Y13A)**
PDB ID	8FJ0	8FK6	8FK8	8FJ2	8FJ3
**Data collection**					
Wavelength (Å)	0.953	0.953	0.953	0.953	0.953
Resolution range	47.11 - 2.89 (3.07 - 2.89)	49.25 - 1.74 (1.77 - 1.74)	48.78 - 1.96 (2.01 - 1.96)	46.34 - 2.07 (2.13 - 2.07)	48.55 – 2.07 (2.13 -2.07)
Space group	P 1 2_1 1	P 4_1 2_1 2	P 4_1 2_1 2	I 2 2 2	P 4_1 2_1 2
Cell dimensions					
a, b, c (Å)	82.4 49.6 88.3	72.3 72.3 67.2	72.5 72.5 65.9	60.6 62.5 92.6	72.2 72.2 65.5
a, b, g (°)	90 112.9 90	90 90 90	90 90 90	90 90 90	90 90 90
R_{merge}	0.427 (2.795)	0.052 (0.351)	0.130 (1.442)	0.210 (1.954)	0.197 (1.411)
R_{meas}	0.465 (3.040)	0.054 (0.358)	0.133 (1.470)	0.219 (2.048)	0.201 (1.440)
R_{pim}	0.180 (1.179)	0.011 (0.069)	0.026 (0.282)	0.062 (0.598)	0.040 (0.285)
I/s(I)	5.4 (1.5)	39.4 (10.4)	18.2 (2.8)	8.7 (2.0)	14.1 (3.4)
$CC_{1/2}$	0.971 (0.475)	0.999 (0.985)	0.999 (0.810)	0.992 (0.551)	0.998 (0.803)
Completeness (%)	96.2 (90.5)	100 (99.4)	100 (99.4)	99.3 (92.7)	99.9 (98.9)
Total reflections	97403 (14332)	489279 (26691)	335070 (23669)	139936 (8894)	272655 (20935)
Unique reflections	14772 (2250)	18937 (1025)	13129 (903)	10995 (785)	11114 (846)
Multiplicity	6.6 (6.4)	25.8 (26.0)	25.5 (26.2)	12.7 (11.3)	24.5 (24.7)
**Refinement**					
R_{work} / R_{free}	0.253 / 0.284	0.206 / 0.223	0.241 / 0.262	0.213 / 0.230	0.213 / 0.229
No. of atoms	2886	1226	1078	1096	1160
Macromolecules	1875	1123	1026	1053	1082
Solvent	11	103	52	43	78
Average B factors	62.5	38.9	42.5	57.2	37.1
Macromolecules	62.5	38.8	42.6	57.4	37.2
Solvent	50.3	39.5	40.0	52.5	36.1
r. m. s. deviations					
Bond lengths (Å)	0.002	0.003	0.018	0.008	0.019
Bond angles (°)	0.70	0.63	1.80	1.02	1.67
Ramachandran statistics (%)					
Favoured	89.40	96.62	96.32	97.10	97.18
Allowed	8.99	3.38	3.68	2.90	2.82
Outliers	1.61	0	0	0	0
Clashscore	4.53	1.37	6.20	5.37	7.22
MolProbity score	2.38	1.08	1.59	1.45	1.55

Supplementary Table 12. MD systems setup

		EVA-A free #1	EVA-A free #2	EVA-P free #1	EVA-P free #2	EVA-A complex	EVA-P complex
Box dimension (Å)	a	48.761	56.878	54.251	58.608	79.051	75.884
	b	46.820	52.558	49.125	49.179	59.505	57.778
	c	62.696	49.685	58.233	57.146	53.992	57.552
Total number of atoms		13752	13830	14623	15397	24061	23702
Total number of water molecules		4185	4211	4438	4696	7240	7083
Other molecules	Cl^-	5	5	2	2	12	9
