# Supplementary material for: Engineering broad-spectrum inhibitors of inflammatory chemokines from subclass A3 tick evasins
Source: Nat Commun. 2023 Jul 14;14:4204. doi: 10.1038/s41467-023-39879-3 (PMC10349104; doi:10.1038/s41467-023-39879-3)
Supplement: Supplementary file 3 — Reporting Summary [file 41467_2023_39879_MOESM3_ESM.pdf]

nature portfolio

Corresponding author(s): Martin J. Stone, Ram Prasad Bhusal

Last updated by author(s): 30/06/23

Reporting Summary

Nature Portfolio wishes to improve the reproducibility of the work that we publish. This form provides structure for consistency and transparency in reporting. For further information on Nature Portfolio policies, see our Editorial Policies and the Editorial Policy Checklist.

Statistics

For all statistical analyses, confirm that the following items are present in the figure legend, table legend, main text, or Methods section.

n/a	Confirmed	
☐	☒	The exact sample size (*n*) for each experimental group/condition, given as a discrete number and unit of measurement
☐	☒	A statement on whether measurements were taken from distinct samples or whether the same sample was measured repeatedly
☐	☒	The statistical test(s) used AND whether they are one- or two-sided<br>*Only common tests should be described solely by name; describe more complex techniques in the Methods section.*
☒	☐	A description of all covariates tested
☐	☒	A description of any assumptions or corrections, such as tests of normality and adjustment for multiple comparisons
☐	☒	A full description of the statistical parameters including central tendency (e.g. means) or other basic estimates (e.g. regression coefficient) AND variation (e.g. standard deviation) or associated estimates of uncertainty (e.g. confidence intervals)
☐	☒	For null hypothesis testing, the test statistic (e.g. *F*, *t*, *r*) with confidence intervals, effect sizes, degrees of freedom and *P* value noted<br>*Give P values as exact values whenever suitable.*
☒	☐	For Bayesian analysis, information on the choice of priors and Markov chain Monte Carlo settings
☒	☐	For hierarchical and complex designs, identification of the appropriate level for tests and full reporting of outcomes
☒	☐	Estimates of effect sizes (e.g. Cohen's *d*, Pearson's *r*), indicating how they were calculated

Our web collection on statistics for biologists contains articles on many of the points above.

Software and code

Policy information about availability of computer code

Data collection	Surface plasmon resonance data collection: Biacore T100 Control Software version 2.0.4<br>cAMP inhibition and ERK phosphorylation assay data collection : PHERAstar FS plate reader version 5.41<br>Crystallographic data collection: qeGUI Eiger<br>Molecular dynamics simulations: Desmond, Protein preparation wizard, and PROPKA are all part of the Schrodinger suite (Schrodinger Release 2022-3)
Data analysis	Sequence and phylogenetic analysis: Alignment: MUSCLE; tree generation: R version 4.1.0, ape 5; visualisation: ggtree 3.2.1; and sequence logo ggseqlogo 0.1<br>Surface plasmon resonance data analysis: Biacore T100 Evaluation Software version 2.0.4<br>cAMP inhibition and ERK phosphorylation assay data analysis: MARS 3.32 and GraphPad Prism 9.0.1<br>Crystallographic data analysis: XDS (version Jan 10, 2022); Auto-Rickshaw 1.8; CCP4 7.1; Phenix 1.18.2-3874; COOT 0.9.6; and PyMOL 2.5.1<br>Molecular dynamics data analysis: visual molecular dynamics (VMD) version 1.9.4<br>Mass spectroscopy data analysis: Data explorer software version 3.4 build 192

For manuscripts utilizing custom algorithms or software that are central to the research but not yet described in published literature, software must be made available to editors and reviewers. We strongly encourage code deposition in a community repository (e.g. GitHub). See the Nature Portfolio guidelines for submitting code & software for further information.

Data

Policy information about availability of data

All manuscripts must include a data availability statement. This statement should provide the following information, where applicable:

- Accession codes, unique identifiers, or web links for publicly available datasets
- A description of any restrictions on data availability
- For clinical datasets or third party data, please ensure that the statement adheres to our policy

The coordinates and structure factors have been deposited in the Protein Data Bank under the following accession codes: EVA-A:CCL7, 7SCU [https://doi.org/10.2210/pdb7SCU/pdb]; EVA-A:CCL11, 7SCS [https://doi.org/10.2210/pdb7SCS/pdb]; EVA-A:CCL16, 7SCT [https://doi.org/10.2210/pdb7SCT/pdb]; EVA-A:CCL17, 7SCV [https://doi.org/10.2210/pdb7SCV/pdb]; EVA-A(Y44A):CCL2, 8FJ0 [https://doi.org/10.2210/pdb8FJ0/pdb]; EVA A(Y44A):CCL7, 8FK6 [https://doi.org/10.2210/pdb8FK6/pdb]; EVA-A(L39P):CCL7, 8SK8 [https://doi.org/10.2210/pdb8SKK/pdb], 8FK8, EVA-A(C8):CCL17, 8FJ2, [https://doi.org/10.2210/pdb8FJ2/pdb]; and EVA-A:CCL7(Y13A), 8FJ3 [https://doi.org/10.2210/pdb8FJ3/pdb].
The data that support the findings of this study are available within the paper and its Supplementary Information files. Source data are provided with this paper.

Human research participants

Policy information about studies involving human research participants and Sex and Gender in Research.

Reporting on sex and gender	Sex- and gender- based analysis are not relevant to this study.
Population characteristics	Population characteristics are not relevant to this study.
Recruitment	Participant recruitment is not relevant to this study.
Ethics oversight	Ethics approval is not relevant to this study.

Note that full information on the approval of the study protocol must also be provided in the manuscript.

Field-specific reporting

Please select the one below that is the best fit for your research. If you are not sure, read the appropriate sections before making your selection.

☒ Life sciences ☐ Behavioural & social sciences ☐ Ecological, evolutionary & environmental sciences

For a reference copy of the document with all sections, see nature.com/documents/nr-reporting-summary-flat.pdf

Life sciences study design

All studies must disclose on these points even when the disclosure is negative.

Sample size	All binding and inhibition experiments were performed three times, independently, in vitro using established methods with high reproducibility (https://doi.org/10.1073/pnas.2122105119). The choice to perform three independent replicates was made to enable calculation of mean and standard error. Similarly molecular dynamics simulations were performed three times, independently to increase sampling of conformations. No sample size calculation was performed for MD simulations. The number of repeat simulations were chosen based on experience and generally accepted standards within MD community. Each MD simulation was initiated with different random seeds to further enhance sampling.
Data exclusions	No data were excluded.
Replication	All binding and inhibition experiments and molecular dynamics simulations were performed three times, independently. In all cases, the standard errors of replicate measurements (for a single experimental condition) were small in comparison to the differences between measurements for different experimental conditions. This is evident from the data points and/or error bars shown in the figures.
Randomization	Randomization is not relevant for this study because there was no allocation of experimental groups. This study uses standard structural biology and in vitro or cell culture protein biochemical assays where randomization is not required.
Blinding	No experiment that would require blinding was performed in this study. Blinding is not a general practice in standard structural biology and in vitro or cell culture protein biochemical assays that is reported in this study.

Reporting for specific materials, systems and methods

We require information from authors about some types of materials, experimental systems and methods used in many studies. Here, indicate whether each material, system or method listed is relevant to your study. If you are not sure if a list item applies to your research, read the appropriate section before selecting a response.

Materials & experimental systems

n/a	Involved in the study	
☒	☐	Antibodies
☐	☒	Eukaryotic cell lines
☒	☐	Palaeontology and archaeology
☒	☐	Animals and other organisms
☒	☐	Clinical data
☒	☐	Dual use research of concern

Methods

n/a	Involved in the study	
☒	☐	ChIP-seq
☒	☐	Flow cytometry
☒	☐	MRI-based neuroimaging

Eukaryotic cell lines

Policy information about cell lines and Sex and Gender in Research

Cell line source(s)	1. FlpIn CHO cells stably expressing the chemokine receptors CCR2 or CCR5 were established in-house from parental FlpIn CHO cells purchased from Thermo Fisher Scientific, using methods described previously (Lim et al., Int. J. Mol. Sci. 2021, 22, 4232. https://doi.org/10.3390/ijms22084232).<br>2. THP-1 cells endogenously expressing the chemokine receptors CCR1 and CCR2 were purchased from ATCC (Manassas, VA).
Authentication	1. FlpIn CHO cells stably expressing the chemokine receptors CCR2 or CCR5 were authenticated by monitoring signalling responses to a suite of chemokine ligands.<br>2.THP-1 cells endogenously expressing the chemokine receptors CCR1 and CCR2 were authenticated by monitoring signalling responses to a suite of chemokine ligands.
Mycoplasma contamination	Cells were not tested for mycoplasma.
Commonly misidentified lines<br>(See ICLAC register)	Commonly misidentified cell lines were not used in this study.
